# Prenatal immune activation and adult Poly(I:C) re-challenge promote neuroimmune priming and AD-related behavioural, cellular and molecular alterations in wild-type mice

**DOI:** 10.3389/fimmu.2026.1845312

**Published:** 2026-07-08

**Authors:** Giacomo Giacovazzo, Valentina Latina, Zuleyha Nihan Yurtsever, Iliana Piccolino, Filomena Iannuzzi, Paola Bossù, Giuseppina Amadoro, Roberto Coccurello

**Affiliations:** 1 Department of Experimental Neurosciences, Istituto di Ricovero e Cura a Carattere Scientifico (IRCSS) Santa Lucia Foundation, Rome, Italy; 2 Institute of Translational Pharmacology, National Research Council, Rome, Italy; 3 European Brain Research Institute (EBRI), Rome, Italy; 4 Laboratory of Experimental Neuropsychobiology, Scientific Institute for Research, Hospitalization, and Healthcare Istituto di Ricovero e Cura a Carattere Scientifico (IRCSS) Santa Lucia Foundation, Rome, Italy; 5 National Research Council (CNR), Institute for Complex System (ISC), Rome, Italy

**Keywords:** Alzheimer’s disease, amyloid beta (Aβ), amyloid precursor protein, cognitive deficit, hippocampus, microglia, motivational blunting, neuroimmune priming

## Abstract

**Objective:**

Alzheimer’s Disease (AD) is neurodegenerative disorder characterized by deposition of Aβ plaques, tau-positive neurofibrillary tangles, neuroinflammation and clinical dementia. Epidemiological and experimental evidence suggest that peripheral immune inflammation is a risk factor for age-related neurodegeneration but whether its sustained activation is sufficient to drive behavioural, molecular and cellular changes consistent with AD-associated neurodegenerative vulnerability remains unclear. Here we investigated whether prenatal immune stimulation followed by an adult systemic re-challenge with Polyinosinic-polycytidylic acid (Poly(I:C)) induces persistent cognitive/motivational/social deficits and hippocampal neurodegeneration in wild-type mice, consistent with long-lasting neuroimmune priming mechanism(s).

**Methods:**

Pregnant C57Bl/6J dams received intravenously Poly(I:C) at gestational day 17 and male offspring received intraperitoneal Poly(I:C) at 9 months (single- or double-hit design) and were analyzed at 12 months. Recognition memory, working memory, reward-related learning, and social interaction were assessed followed by hippocampal Western blotting and immunofluorescence.

**Results:**

Poly(I:C)-exposed mice exhibited impaired recognition and working memory, reduced palatable food-induced conditioned place preference, and blunted social investigation. These behavioral abnormalities were accompanied by increased amyloidogenic APP processing (BACE1/PSEN1 upregulation and β-CTF accumulation), tau dysregulation (AT8 hyperphosphorylation), microglial activation (Iba1/CD68 upregulation and process retraction), synaptic alterations (α-synuclein reduction), and bioenergetic impairment (reduced mitochondrial and glycolytic markers) in the hippocampus.

**Conclusion:**

Overall, these findings indicate that repeated prenatal and postnatal peripheral activation of innate immunity may act as a contributing factor to neurodegenerative phenotype with features relevant to AD-related susceptibility, paving the way for the development of next-generation therapeutical interventions affecting systemic-to-brain inflammatory signaling.

## 1 Introduction

The breakdown of neuroimmune homeostasis has been associated with the pathogenesis of neurodegenerative disorders, including Alzheimer’s disease (AD) which is a multifaceted disorder characterized by neuroinflammation, β-amyloid (Aβ) plaques, tau-positive neurofibrillary tangles, and progressive memory decline (1, 2). Genome-wide association studies (GWAS) in humans, together with preclinical findings in animal models, have demonstrated that AD is not merely a neurocentric disorder but a systemic condition triggered by a reciprocal interplay between the periphery and the brain, involving peripheral immune dysregulation and neuroinflammation (3–6). A neuroimmune axis has been proposed as a key driver of AD etiology, in which bidirectional central-peripheral interactions critically impact on the disease onset and progression (7, 8). In particular, the so-called “infection hypothesis of AD” posits that immune challenge following a systemic inflammation triggers a self-perpetuating cascade of neurotoxic protein aggregation and neuroinflammation culminating in neuronal death and clinical dementia (9). An antimicrobial role of Aβ acting as effector molecule of innate immunity in protection against infection has been also proposed (10). Viral-like immune insults may act as “priming” events for the innate immune system, promoting long-lasting changes in inflammatory responsiveness that, in combination with additional vulnerability factors such as aging, may contribute to a state of chronic low-grade inflammation. Such persistent immune dysregulation has been implicated in several conditions associated with increased risk of sporadic AD, including brain trauma, obesity, physiological aging, systemic inflammation, and bacterial or viral infection (1, 2, 11–17). A “primed” status, characterized by robust upregulation of inflammatory programs in innate immune cells of both the brain and periphery, has been described in AD (18–20). Elevated levels of proinflammatory cytokines and enhanced inflammatory response are detected in blood monocytes from AD patients (21–24) in correlation with brain atrophy and rapid conversion from Mild Cognitive Impairment (MCI) to more severe stage of full-blown dementia (25).

Microglia, the brain-resident macrophages, in AD mouse models and human AD patients appear to be hyperactivated and to display an altered reactivity threshold, producing increased amounts of pro-inflammatory mediators in response to secondary systemic inflammation (26, 27). In line with previous reports (28–30), the peripheral, repeated injections of bacterial endotoxin LipoPolySaccharide (LPS), a Pathogen-Associated Molecular Pattern(s) (PAMPs) routinely used to study the brain’s response to systemic immune challenges (31, 32), evokes a primed immune state in microglia from APP23 (APP751Swedish double mutation (K670M/N671L)) AD mice and significantly influences their neuropathology later in life by increasing cerebral β-amyloidosis (33). Interestingly, an elevation in levels of both Aβ1–40 and Aβ1–42 along with IL-1β protein production following LPS administration occurs in the brains of 16- but not 6-month-old Tg2576 (APP695Swedish double mutation (K670M/N671L)), indicating that Aβ plaques accumulating with aging in this APP-overexpressing AD transgenic mouse model sensitize microglial cells and that this hyperactivation state under pro-inflammatory conditions contributes or even accelerates the extent and the timing of neurodegenerative process (34). Consistent with this finding, an amplified inflammatory cytokine response to peripheral inoculation of LPS with exacerbated production of IL-1β and inducible Nitric Oxide Synthase (iNOS) is also detected in the brains of another transgenic mouse line carrying the AD familial M146V PS1 mutation and showing progressive Aβ deposition as compared to wild-type mice (35). With respect to neuronal survival and memory performance, recurrent inflammatory insult eliciting disproportionate and hypersensitive responses with elevated IL-1β and IL-6 in microglia from amyloid-laden brains of APP/PS1 AD mice has been reported to drive neuronal death and cognitive impairment (36). Finally, an increased site-specific tau hyperphosphorylation is visible in brains from 3xTg (APPK670, N/M671L, PS1M146V and tauP301L) AD mice (37) following chronic delivery of LPS, indicating that sustained systemic inflammation also negatively impacts on tau metabolism. Nevertheless, althought these findings support the existence of a causal link between systemic inflammation and brain neurodegeneration, whether systemic innate immune activation can operate as a contributing factor to the onset of neurodegenerative processes with features relevant to AD remains incompletely defined. To study how the repeated challenge of innate immunity may be partly responsible for behavioural and neurochemical alterations associated with AD phenotype, we used the Poly(I:C) mouse model (38), based on maternal delivery (Maternal Immune Activation, MIA) of a synthetic analogue of viral dsRNA such as Polyinosinic-polycytidylic acid (i.e. Poly(I:C)) (32, 39) which provokes a cytokine-linked acute inflammatory response both in maternal and fetal brains (40–42). The biological effects of Poly(I:C) are mainly initiated by binding to Toll-like receptor 3 (TLR3) (43, 44) expressed by circulating macrophages and dendritic cells whose stimulation triggers an innate immune response via transcriptional activation of pro-IL-1β and pro-IL-18 that are then processed into their active mature forms by inflammasome (45). According to the “double-hit model”, after prenatal infection with viral mimetic Poly(I:C) (“first hit”) during a time window (GD17) of enhanced susceptibility to inflammatory cytokines (46, 47), a secondary exposure of offspring to this immunostimolant at 9 months of age (“double hit”) generates long-lasting neuroimmune priming linked with the development of neurodegenerative diseases (41, 48). Therefore, with the intent of investigating how deregulation of innate immunity can contribute to CNS vulnerability, we assessed in this experimental animal model selected molecular and cellular pathways relevant to AD-associated neurodegeneration, including APP processing, tau phosphorylation, synaptic markers, mitochondrial and glycolytic proteins, together with behavioral (cognitive and non-cognitive) outcomes.

## 2 Materials and methods

### 2.1 Animals and ethical approval

All animal experiments complied with the ARRIVE guidelines and were carried out in accordance with the ethical guidelines, the European Council Directive (2010/63/EU) and the Italian Animal Welfare legislation (D.L. 26/2014). Experimental approval was obtained from the Italian Ministry of Health (Authorization n. 285/2023-PR). This study was carried out according to the principles of the 3Rs (Replacement, Reduction and Refinement) to minimize animal suffering and to reduce the number of animals used.

### 2.2 *In vivo* immune challenge

Animal treatment was carried out on the basis of the protocol described by (48), with *in utero* infection with Poly(I:C), followed by a secondary immune challenge in adulthood offspring. In detail, Poly(I:C) potassium salt (PolyI:C) (at 5 mg/kg dose dissolved in sterile 0.9% NaCl; cat. no. P9582 Sigma-Aldrich) or vehicle (V) (0.9% NaCl) were intravenously (i.v.) administered to pregnant 4-month-old C57Bl/6J mice during prenatal stage, at late gestational day 17 (GD17) a time window of enhanced vulnerability to inflammatory cytokines (46, 47) corresponding to the second-to-third trimesters of human pregnancy with respect to developmental biology and percentage of gestation from mice to human (49). The 5 mg/kg dose was chosen according to the previously-published double-hit Poly(I:C) paradigm to induce a robust, reproducible viral-like innate immune activation (48). This regimen should be interpreted as a controlled experimental immune challenge rather than a direct model of naturally occurring viral infection during pregnancy. Late gestation maternal immune activation induces a penothype associated with hippocampal cognitive and neuropathological changes in adults (41, 48). A secondary immune Poly(I:C) challenge (PolyI:C) or vehicle (V) were subsequently administered intraperitoneally (i.p.) to male offspring at 9 months of age (9M) corresponding to mature adulthood. This time point was selected to be consistent with the well-established double-hit Poly(I:C) model as previously-described by (48) in which a late-gestational immune challenge (GD17) followed by a secondary systemic Poly(I:C) re-challenge in mature adulthood (9M) promoted AD-relevant neuropathological alterations in aging wild-type mice. Besides, since we aimed to evaluate behavioral, biochemical, and morphological outcomes at 12 months, offspring were administered with the secondary Poly(I:C) challenge three months before endpoint analyses (i.e. at 9 months). This regimen allowed us to test whether an immune re-challenge during mature adulthood could accelerate or unmask neurodegenerative-like alterations just at the onset of age-related declines rather than in advanced aging.

**Figure 1:**
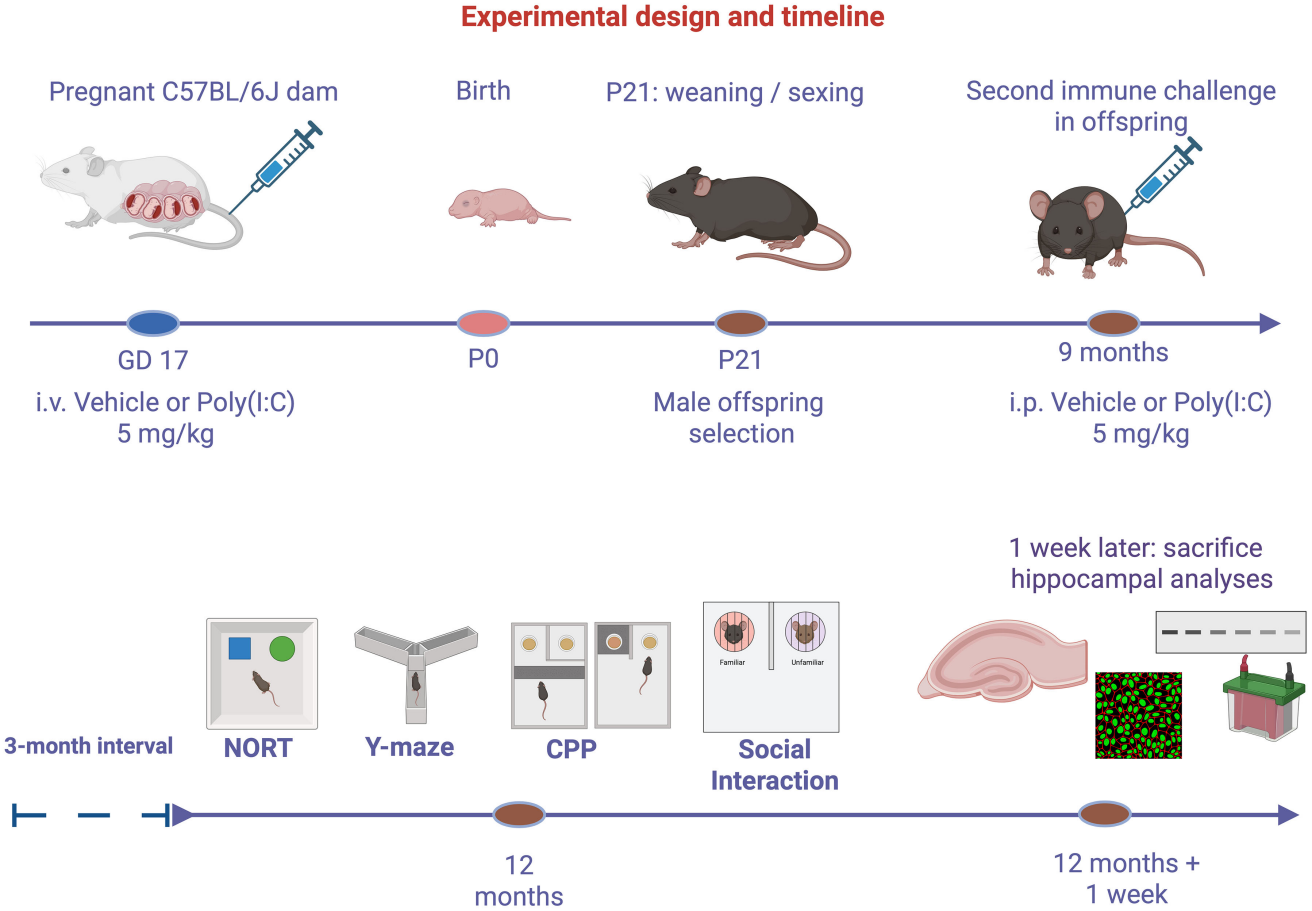
Experimental design and timeline. Schematic depicting the double-hit Poly(I:C) paradigm and the experimental timeline of behavioral, biochemical and morphological assessments. Pregnant 4-month-old C57BL/6J dams were intravenously administered with vehicle or Poly(I:C) (5 mg/kg) at gestational day 17 (GD17). Male offspring were selected at postnatal day 21 (P21) and were intraperitoneally injected with vehicle or Poly(I:C) (5 mg/kg) at 9 months of age (9M). After an interval of 3 month (at 12 months, 12M), the same cohort of male mice underwent the behavioral battery, including Novel Object Recognition Test (NORT), Y-maze spontaneous alternation, Conditioned Place Preference (CPP), and social interaction. One week after completion of behavioral testings, animals were sacrificed for hippocampal biochemical (Western blotting) and morphological (Immunofluorescence) analyses. This drawing was created with BioRender.com.

According to our experimental design, mice were randomized into four experimental groups: 1) V GD17/V 9M (V/V); 2) VGD17/PolyI:C 9M (V/PolyI:C); 3) PolyI:C GD17/V 9M (PolyI:C/V); 4) PolyI:C GD17/PIC 9M (PolyI:C/PolyI:C). Three months after the last injection, 12-month-old animals underwent behavioural testing or were sacrificed for brain removal and tissue collecting, biochemical and morphological analyses (**Figure 1**).

### 2.3 Animal, housing, handling and allocation

C57BL6/J mice were purchased from Charles River and housed in polycarbonate cages (29.5 × 13 × 11.5 cm) under normal light/dark cycles (12h/12h, light off at 8 PM) and standard laboratory conditions (temperature 21+/- 3 °C; humidity 50 +/- 10%) with *ad libitum* access to food and water. After mating and formation of vaginal plug (GD0), pregnant females were housed individually until GD17 when they were randomly assigned to Poly(I:C) or saline group. After injection, pregnant dams were immediately returned to their home cage and left undisturbed until 7 days after birth of offspring when hut and nesting material were changed. Pups born to Poly(I:C)- and vehicle-treated mothers were weaned and sexed on postnatal day 21 (PN21) and litters of the same sex were kept in separate cage (4–5 animals/cage) until adulthood. Only male mice were used to avoid confounding factors due to hormonal fluctuation of sexual dimorphism. To minimize potential litter effects, offspring from different litters were distributed across experimental groups, and no more than one or two animals per litter were randomly assigned to the same experimental condition whenever feasible. Animals were monitored once daily throughout the experiment and whenever signs of suffering manifest (loss of body weight, hunched posture any other behavioral indicators of pain or distress) they were immediately euthanized and not included in the subsequent experimental analyses. Animals underwent behavioural testing were sacrificed after one-week interval to avoid bias due to task-related stress.

### 2.4 Cognitive assessment

#### 2.4.1 Novel object recognition test

Recognition memory was evaluated using the Novel Object Recognition Test (NORT) in a circular Plexiglas arena (60 cm diameter, wall height 50 cm) with a white floor divided into 25 equal squares by black lines. The objects were placed 15 cm away from the walls. The distance between the two objects during both the pre-test and test phases was 30 cm. The arena was indirectly illuminated, and a striped card placed on one wall served as a distal visual cue. The test was performed according to a previously described protocol (50, 51) with minor modifications. During the habituation phase, mice were allowed to explore the arena for 20 min, and baseline locomotor activity was recorded. After a 5-min interval, mice were returned to the arena for a 10-min training session in the presence of two identical objects placed in the center. After 24 h, each mouse was reintroduced into the arena for an 8-min test session, during which one familiar object was replaced with a novel object. The position of the novel object was randomized between the left and right sides. Exploration was recorded by video and defined as the mouse placing its nose within 1 cm of the object. The preference index (PI) was calculated as [(time spent exploring the novel object − time spent exploring the familiar object)/(time spent exploring the novel object + time spent exploring the familiar object)] × 100, as previously described (50, 51).

#### 2.4.2 Y-maze spontaneous alternation

Short-term spatial working memory was assessed using the spontaneous alternation Y-maze task in the absence of reward or punishment (52, 53). The apparatus consisted of a black Plexiglas Y-maze with three arms (A, B, and C), each measuring 30 cm in length, 10 cm in width, and 20 cm in height. Each arm contained a distinct visual cue. Mice were habituated to the testing room for 30 min before testing. At the start of the session, each mouse was placed in the center of the maze and allowed to explore freely for 8 min. An arm entry was scored when all four paws entered the same arm. The sequence and total number of arm entries were recorded using a video-tracking system. A spontaneous alternation was defined as three consecutive entries into three different arms (i.e., ABC, ACB, BAC, BCA, CAB, or CBA). Mice with fewer than 8 arm entries during the 8-min session were excluded from the analysis. The percentage of spontaneous alternation was calculated as the number of successful triads divided by the total number of arm entries minus two, multiplied by 100.

### 2.5 Motivational and social behavior

#### 2.5.1 Conditioned place preference

As previously published (54, 55), palatable food-induced Conditioned Place Preference was evaluated in a custom-built two-chamber Plexiglas apparatus composed of two compartments (15 × 15 × 20 cm each) connected by a central corridor (15 × 5 × 20 cm) with sliding doors (4 × 20 cm) allowing controlled access. The compartments were distinguished by different visual and tactile patterns on the walls and floors and by distinct arrangements of two black Plexiglas triangular prisms (5 × 5 × 20 cm), which served as contextual cues. Illumination and environmental conditions were balanced between chambers to reduce any intrinsic side bias. For the pre-conditioning session, each mouse was placed in the central corridor and allowed to freely explore both compartments for 15 min in the absence of food. Time spent in each chamber was recorded to assess baseline preference. The conditioning phase consisted of six consecutive daily sessions. During each 30-min session, mice were alternately confined to one chamber paired with palatable food reward (0.5 g milk chocolate; Milka Alpine Milk Chocolate, 5.31 kcal g⁻¹) or to the other chamber paired with regular chow (RC; Mucedola 4RF21 diet). The chocolate-paired chamber was defined as the paired compartment, whereas the chow-paired chamber was defined as the unpaired compartment. Food portions were adjusted to maintain isocaloric conditions across sessions. Chocolate consumption was measured in all groups during conditioning. Animals were randomly assigned to treatment conditions and experimenters were blinded to group allocation during behavioral scoring and analysis. Moreover, within each genotype, the association between food type and chamber-specific cues was counterbalanced. On the post-conditioning test day, mice underwent the same 15-min free-exploration session used at baseline, and the time spent in each chamber was recorded as a measure of conditioned preference. Behavioral activity was acquired using a CCD video camera and analyzed with EthoVision XT software (Noldus Information Technology). CPP preference scores were derived from the time spent in each compartment during the pre- and post-conditioning sessions. Chocolate intake was calculated by weighing the remaining food after each chocolate-paired session and averaging the values obtained across the conditioning period.

#### 2.5.2 Social interaction

Social preference was evaluated using a modified three-chamber social interaction test designed to assess the ability of mice to discriminate between social and non-social stimuli. The apparatus consisted of a Plexiglas box divided into three interconnected chambers of equal size, with openings allowing free access between compartments. Mice were habituated to the testing room for 120 min before testing. During the initial habituation phase, each mouse was placed in the central chamber and allowed to freely explore the entire apparatus for 10 min. After 24 h, mice underwent a non-social novelty exploration session lasting 5 min. In this phase, two identical wire cages were placed in the lateral chambers, with a novel object positioned inside one cage and the other left empty. After an additional 24-h interval, mice were subjected to the social preference session. During this phase, an unfamiliar age- and sex-matched conspecific was placed inside one wire cage, whereas an inanimate object of comparable size was placed inside the other. The position of the social and non-social stimuli was counterbalanced between the left and right chambers to avoid side bias. Each mouse was then returned to the central chamber and allowed to freely explore the apparatus for 5 min. This paradigm was used to assess non-social novelty exploration during the intermediate session and social preference during the final session. Behavioral activity was video recorded, and the time spent in each chamber as well as the time spent investigating each stimulus were quantified. Exploration (sec) was defined as direct nose-oriented investigation of the wire cage at close proximity, whereas climbing or sitting on the cage was not considered active investigation. Social preference was expressed as the relative time spent investigating the social stimulus versus the non-social stimulus.

### 2.6 Tissue collection, cryopreservation and preparation

For biochemical analysis, after completion of all the tasks included in the cognitive assessment, animals from four experimental groups (V/V, V/PolyI:C, PolyI:C/PolyI:C, PolyI:C/V) were sacrificed by cervical dislocation to avoid anesthesia-mediated tau phosphorylation. Brains were collected, the meninges were carefully removed and dissected hippocampi are immediately snap-frozen on dry-ice and stored at -80 °C until use. Frozen hippocampi were diced and homogenized in ice-cold RIPA buffer (50 mM Tris-HCl pH 8, 150 mM NaCl, 1% NP40, 0.1% SDS, 0.5% sodium deoxycholate) plus proteases inhibitor cocktail (Sigma-Aldrich, P8340) and phosphatase inhibitor cocktail (Sigma-Aldrich, P5726/P2850) for 30 min and then centrifuged at 4 °C for 20 min at 12,000 rpm. The amount of total protein extracts was determined by Bradford assay (Protein Assay Dye Reagent Concentrate, Bio-Rad, Hercules, CA, USA) as previously reported (52). For morphological analysis (51), animals were sacrificed by intraperitoneal overdose of anesthetic and were transcardially perfused with ice-cold PBS pH=7.4 to remove blood contamination. Then the brains were carefully removed from the skull, post-fixed in 4% ParaFormAldehyde (PFA) solution in PBS overnight at 4 °C and, then, were passed into 30% sucrose solution in PBS for 48–72 hrs at 4 °C until equilibration before cryosectioning. The brains were frozen by immersion in ice-cold isopentane for 3 min before being sealed into vials and stored at -80 °C until use.

### 2.7 Western blot analysis and semi-quantitative densitometry

Equal amounts of protein extracts (150µg) were loaded for each blot and size-fractionated by ProteinEle Precast Tris-Glicine gels 4-20% (TransGen Biotech Co., LTD) according to (52). The filters were blocked in TBS-T containing 4% non-fat dried milk for 1h at room temperature or overnight at 4 °C. Proteins were visualized using appropriate primary antibodies. All primary antibodies were diluted in TBS and incubated with the nitrocellulose blot overnight at 4 °C. Incubation with secondary peroxidase coupled anti-mouse, anti-rabbit (anti-mouse or anti-rabbit, Sigma-Aldrich, diluted 1:8:000) was developed by using the Enhanced ChemiLuminescence western blotting immunodetection system (ECL) (Thermo Scientific SuperSignal West Pico, 34080; Amersham ECL Prime, RPN2232) and, then, the signal was detected by using the iBright Imaging Systems (Thermo Fisher Scientific). For statistical analysis, β-actin was used as internal control of protein loading for all proteins of interest and semi-quantitative densitometric analysis was carried out by using ImageJ 1.4 (https://imagej.net/ij/). For quantification, we measured the band intensity by using a signal in the linear range. Appropriate counterbalancing during gel loading and running technical replicates were carried out to improve reproducibility. For immunoblot normalization, β-actin was used as loading control. Whenever possible, β-actin was detected on the same gel/membrane of the protein of interest, provided that the 42-kDa β-actin band was clearly distinguished from the target protein band. In other cases, including molecular weight overlap or experiments in which multiple proteins were analyzed from the same collection of samples, equal amounts of total protein were run in parallel under identical electrophoretic and transfer conditions. One membrane was probed for the protein of interest, whereas the corresponding membrane was probed for β-actin. This approach was used to avoid repeated stripping/reprobing of the same membrane, which may affect signal intensity and introduce technical variability. Control for nonspecific binding of the secondary antibodies was performed by omitting the primary antibody (**Supplementary Figure 1**). The following antibodies were used: anti-Amyloid Precursor Protein 22C11 (66–81aa of N-terminus) mouse APP-MAB348 Chemicon; anti-BACE1 (clone 61-3E7) mouse sc-33711 Santa Cruz; anti-Presenilin-1 (clone PS1-loop) mouse MAB5232 Merk Millipore; anti-Neprilysin (clone F4P6H) rabbit 58145 Cell Signaling; Anti-Amyloid Precursor Protein C-Terminal antibody rabbit 8717 Sigma-Aldrich; anti-Glial Fibrillary Acidic Protein (GFAP, clone 2E1) mouse sc-33673 Santa Cruz; anti-Ionized calcium-Binding Adaptor molecule 1 (Iba1) rabbit 019–19741 Wako; anti-PostSynaptic Density Protein 95 (PSD95) rabbit 2507 Cell Signaling; anti-syntaxin 1 mouse S1172 Sigma-Aldrich; anti-Synaptosomal-Associated Protein 25kDa (SNAP25, clone SMI 81) mouse 836301 BioLegend; anti-α synuclein antibody (clone 42) mouse 610,786 BD Transduction Laboratories; anti-Neuronal nuclei (NeuN) antibody (clone A60) mouse MAB377 Millipore; poly-tau (243-441aa) rabbit A0024 Dako; anti-phospho-tau (AT8 p^+^Ser202/Thr205p) mouse MN1020 ThermoFisher Scientific; anti-non-phosho-Tau Tau1 (clone PC1C6, p^-^Ser195/Ser198/Ser199/Ser202) mouse MAB3420 Merck Millipore; anti-Voltage-Dependent Anion Channel (VDAC)/Porin antibody rabbit ab34726 Abcam; anti-OxPhos Complex IV (CoxIV) mouse A21348 Molecular Probes; anti- Translocase of Outer Mitochondrial Membrane 20 (Tom20, FL-145) rabbit sc-11415 Santa Cruz; anti-Cytochrome C (136F3) rabbit 4280 Cell Signaling Technology; anti-pyruvate kinase M2 (PKM2) rabbit 3198 Cell Signaling; anti 6-Phosphofructo-2-Kinase/Fructose-2,6-Biphosphatase 3 (PFKFB3, clone D7H4Q) rabbit 13123 Cell Signaling; anti-β actin mouse S3062 Sigma-Aldrich; anti-mouse IgG (whole molecule)-Peroxidase antibody A4416 Sigma-Aldrich; anti-rabbit IgG (whole molecule)-Peroxidase antibody A9169 Sigma-Aldrich.

### 2.8 Immunofluorescence assays and analyses

For immunofluorescence assays, frozen brains were embedded in Optimal Cutting Temperature compound (OCT) and mounted on cryostat (Laica CM1860 UV) to obtain hippocampal free-floating coronal sections of 20μm thickness according to the guidelines of Allen brain Atlas (https://portal.brain-map.org/anatomy).

After three washes in PBS, the sections were incubated in blocking/permeabilizing solution (10% NGS, 5% BSA, and 0.5% Triton X 100 in PBS pH 7.4, 6 hrs at room temperature) and then probed o.n. at 4 °C with specific primary antibodies (anti- Ionized calcium-Binding Adaptor molecule 1 (Iba1) rabbit 019–19741 Wako, diluted 1:3000; anti-Cluster of Differentiation 68 (CD68) mouse MCA1957GA Bio-Rad, diluted 1:500; anti-Cluster of Differentiation 45 (CD45) rabbit 10558 Abcam, diluted 1:500) in PBS buffer containing 5% NGS, 2.5% BSA and 0.3% Triton X 100. After three washes in PBS buffer with 0.1% Triton X 100, the sections were incubated for 1.5 hrs at room temperature with the specific secondary antibodies (goat anti-rabbit or goat anti-mouse, 488, Jackson ImmunoResearch, Europe Ltd., Sufolk, UK, diluted 1:500) in PBS buffer containing 2.5% NGS, 1.25% BSA and 0.15% Triton X 100. After three washes in PBS buffer with 0.1% Triton X 100, stained slices were mounted on positively charged microscope slides (Bio-Optica, Milan, Italy) and, then, nuclei counterstaining was performed by using fluoroshield mounting medium with DAPI (F6057, Sigma-Aldrich, St. Louis, MO, USA). After coverslipping, microscope slides were stored at 4 °C protected from light until imaging. All incubations were performed under gentle agitation on a laboratory rocket. In preliminary pilot experiments, immunofluorescence analyses were carried out on whole hippocampus but the relative differences among the four experimental groups turned out to be similar throughout the different subfields (CA1, CA2, CA3, DG) so that, in order to be consistent with results from behavioural tasks, representative images were taken from CA2 that is a key subfield critically involved in acquisition of both learning/memory and social behavior. Images (20X) are representative of at least three independent experiments and were acquired with spinning disk system for fast fluorescence confocal microscopy, with led or laser light source, Crest Optics, (Crisel Instruments, Rome, Italy). The acquisition settings for laser power and detector gain were standardized for each analyzed marker and consistent thresholding parameters were applied throughout the study to all images for each stain. For each staining mentioned above, at least 5 randomly selected sections from brains of four experimental groups were analysed. Z-stacks images (n=11) were captured at 0.5 μm intervals with a pinhole of 1.0 Airy unit. Analyses were performed in sequential scanning mode to rule out cross-bleeding between channels. Fluorescence quantification was performed with ImageJ 1.4 software1 by dividing each measured % area (coverage value) of the signal intensity by the total number of DAPI^+^ cells present in all analysed field of view. All counts were performed automatically on each slide using a custom counting macro of ImageJ. For the determination of neurites length, a plugin of ImageJ 1.4.1 (NeuronJ) was used by drawing a line from the center of each soma to the tip of the longest process. For the evaluation of cell body compactness, the soma area and perimeter of Iba1-positive microglial cells were measured using ImageJ 1.4.1 and the cell body compactness was calculated as Area/Perimeter². For the coverage % area, the ROI was selected manually and the area covered by Iba1-positive microglia normalized to the ROI area X100 was calculated with ImageJ 1.4.1 software. Control for nonspecific binding of the secondary antibody was performed by omitting the primary antibody (**Supplementary Figure 1**).

### 2.9 TNF−ɑ ELISA assay on hippocampus and plasma

An *in vitro* whole blood stimulation was carried out by diluting 1:2 whole blood in RPMI medium and stimulating with 2.5 µg/mL R848 (Resiquimod), a specific TRL7/8 agonist, for 18 hours at 37 °C. This is an optimized test to measure functional responsiveness of peripheral immune cells based on their cytokine production since it offers two main advantages: (1) isolates cell-intrinsic responsiveness from confounding systemic factors (such as clearance rates, hormone fluctuations, or tissue sequestration) that affect plasma cytokine levels; and (2) increases sensitivity to detect changes in immune cell priming or tolerance by providing a standardized, reproducible stimulus and incubation period. After incubation, samples were centrifuged at 1000× g (no brake) for 10 minutes at RT and the diluted plasma was collected for ELISA assay.

Hippocampal tissues were lysed in ice cold RIPA buffer supplemented with phosphatase and protease inhibitors, homogenized by sonication, incubated on ice for 20 minutes and, then, centrifuged at 14,000 × g for 30 minutes at 4 °C. The soluble protein fraction was collected and total protein concentration was determined by BCA assay.

TNF−α levels were measured using the DY410 ELISA kit (R&D Systems), according to the manufacturer’s instructions. Briefly, plates were coated with capture antibody, washed, blocked and, then, loaded with samples or standards (100 µL of diluted plasma or 100 µL of hippocampal total protein were applied per well). After incubation, wells were incubated with detection antibody and streptavidin−HRP with washes between steps. A chromogenic substrate was added, the reaction was stopped with acid, and absorbance was read at 450 nm.

### 2.10 Data management and statistical analysis

Values were from at least three independent experiments (n=3) and statistically significant differences were calculated by parametric one-way or two-way analysis of variance (ANOVA) followed by Bonferroni’s *post-hoc* test for multiple comparisons among more than two groups. p<0.05 was accepted as statistically significant (*p<0.05; **p<0.01; ***p<0.0005; ****p<0.0001). Data distribution was assessed for normality utilizing the Shapiro-Wilk test. All experiments were performed whenever feasible, under blinded conditions with the experimenter unware of treatment conditions. Sample size was determined *a priori* using G*Power (version 3.1.9.4) analysis to achieve a statistical power of at least 80% (α = 0.05), based on expected effect sizes derived from preliminary data. Differences among the compared means ≥30% with SD ≤ 20% of the mean within groups were considered to obtain a power of at least 80% with an alpha level of 0.05. Outliners were calculated by Grubbs’ test (Z=mean-value/SD) and excluded when Z is greater than 1.96. Exclusion criteria were applied consistently across the four experimental groups. Unequal sample size with equal variances among the four experimental groups was applied in compliance with the assumption of ANOVA statistical analyses. All statistical analyses were performed using GraphPad Prism 8 software. **Table 1** reports data structure and statistical design for all the analyses performed.

**Table 1:** Statistical design and data structure for behavioral, biochemical and immunofluorescence analyses.

Assay	Experimental unit/data structure	Group size	Statistical analysis	Notes
Novel Object Recognition Test (NORT)	One value per animal Normal distribution	V/V: N = 12; V/Poly(I:C):N=7 Poly(I:C)/V: N=11 Poly(I:C)/Poly(I:C):N=26	One-way ANOVA Shapiro–Wilk normality test Bonferroni post-hoc test	Behavioral analysis performed on individual animals
Y-maze spontaneous alternation	One value per animal Normal distribution	V/V: N= 12 V/Poly(I:C):N=7 Poly(I:C)/V:N=11 Poly(I:C)/Poly(I:C):N =26	One-way ANOVA Shapiro–Wilk normality test Bonferroni post-hoc test	Animals with fewer than 8 arm entries were excluded according to predefined criteria
Conditioned Place Preference (CPP)	One value per animal Normal distribution	V/V: N = 12 V/Poly(I:C):N=7 Poly(I:C)/V:N=11 Poly(I:C)/Poly(I:C):N =26	One-way ANOVA Shapiro–Wilk normality test Bonferroni post-hoc test	CPP score calculated from pre- and post-conditioning compartment preference
Three-chamber social interaction test	One value per animal for each stimulus condition Normal distribution	V/V: N = 12 V/Poly(I:C):N=7 Poly(I:C)/V:N=11 Poly(I:C)/Poly(I:C):N=26	Two-way ANOVA Shapiro–Wilk normality test Bonferroni post-hoc test	Main factors: treatment group and stimulus type
Western blotting (WB)	One value per animal/hippocampal lysate Normal distribution	Exact N varied according to protein marker and sample availability reported figure legend	One-way ANOVA Shapiro–Wilk normality test Bonferroni post-hoc test	Samples were excluded only when predefined technical quality criteria were not met
Immunofluorescence (IF)	One value per animal Multiple sections averaged within each animal Normal distribution	Exact N for protein each protein marker reported in figure legend	One-way ANOVA Shapiro–Wilk normality test Bonferroni post-hoc test	Section-level measurements were averaged per animal before statistical analysis

Data distribution was assessed using the Shapiro-Wilk test. Variance homogeneity was evaluated before applying ANOVA. Parametric analyses were performed only when assumptions were considered appropriate. Exact sample sizes for biochemical and immunofluorescence analyses are reported in the corresponding figure legends.

## 3 Results

### 3.1 Poly(I:C) immune challenge and impact on cognitive and motivational/social behavior

**Figure 2:**
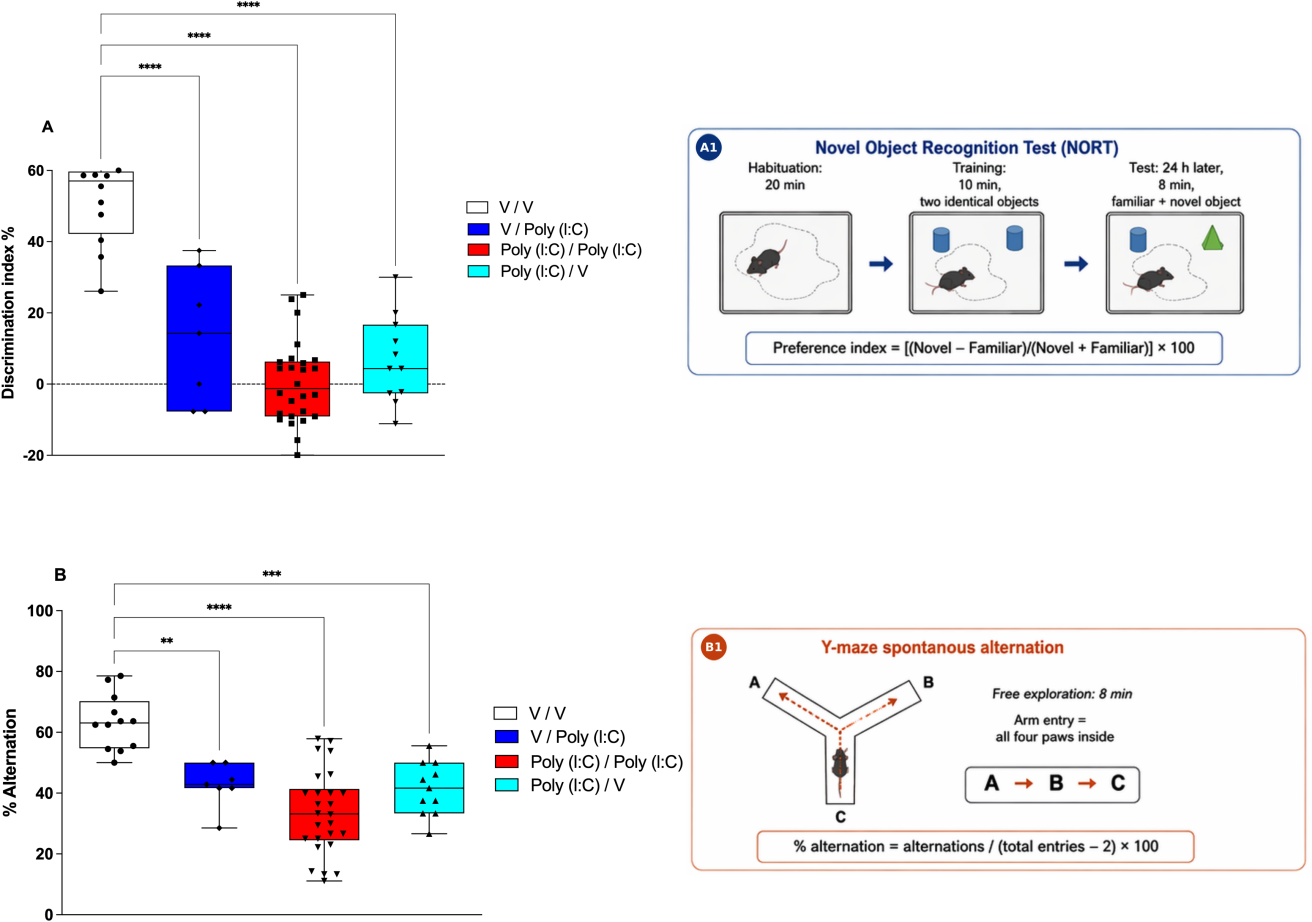
Performance in novel object recognition test (NORT) and Y-maze spontaneous alternation. Prenatal and/or adult exposure to Poly(I:C) induces cognitive impairment in recognition and working memory. **(A)** Novel object recognition test (NORT). Control V/V mice displayed a robust discrimination index, whereas Poly(I:C)-exposed groups (V/PolyI:C, PolyI:C/V and PolyI:C/PolyI:C) showed reduced novel object discrimination, with the largest impairment in the double-challenged PolyI:C/PolyI: Cgroup, approaching chance-level performance (one-way ANOVA, F_(3,52)_ = 40.13, P<0,0001). (A1) Schematic illustration of the Novel Object Recognition Test (NORT) behavioral paradigm. NORT consisted of habituation, training phase with two identical objects followed by a test session 24 h later in which animals were exposed to two different object: one familiar from the training phase and one novel object. This drawing was created with BioRender.com. **(B)** Y-maze spontaneous alternation. Control V/V mice exhibited the highest alternation percentage in contrast to all Poly(I:C)-treated groups showed decreased spontaneous alternation, with the most pronounced deficit in the double challenged PolyI:C/PolyI:C group (one-way ANOVA, F_(3,52)_ = 18.90, P<0,0001). (B1) Schematic illustration of the Y-maze spontaneous alternation behavioral paradigm. The Y-maze assessed spontaneous alternation of animals during 8 min of free exploration in a Y-maze apparatus with three arms. This drawing was created with BioRender.com. Data are presented as box-and-whisker plots with individual data points, and the horizontal line representing the median of the values. Data were analyzed by one-way ANOVA, Bonferroni’s *post hoc* test (**p<0.01; ***p<0.0005; ***p<0.0001). (V/V N = 12; V/PolyI:C N = 7; PolyI:C/V N = 11; PolyI:C/PolyI:C N = 26; **p<0.01; ***p<0.0005; ****p<0.0001).

Novel object recognition memory was significantly affected among the four experimental groups (V/V, V/PolyI:C, PolyI:C/V and PolyI:C/PolyI:C). As shown in **Figure 2A**, control V/V mice exhibited a robust discrimination index, indicating preserved recognition and memory of the novel object. By contrast, mice exposed to Poly(I:C) showed significantly reduced discrimination performance. The V/PolyI:C, PolyI:C/V and PolyI:C/PolyI:C groups displayed all lower discrimination index values than controls, with the PolyI:C/PolyI:C group showing the strongest impairment and values approaching chance performance (one-way ANOVA, Bonferroni’s *post-hoc* test; **p<0.01; ***p<0.0005; ****p<0.0001). No significant differences in the distance travelled during the habituation session were observed among the four experimental groups, indicating comparable baseline locomotor activity (One-way ANOVA analysis Bonferroni’s *post-hoc* test; p>0.9999)(**Supplementary Figure 2**). These data support the conclusion that Poly(I:C) exposure disrupts recognition memory in Poly(I:C)-challenged animals.

Performance in the Y-maze spontaneous alternation task differed markedly among the four cohorts. As shown in **Figure 2B**, control (V/V) mice exhibited the highest alternation percentage, consistent with a, intact short-term spatial working memory. In contrast, mice exposed to Poly(I:C) displayed a significant reduction in spontaneous alternation. This impairment was evident in the V/PolyI:C, PolyI:C/V and PolyI:C/PolyI:C groups relative to controls, with the PolyI:C/PolyI:C group showing the lowest alternation rate (one-way ANOVA, Bonferroni’s *post-hoc* test; **p<0.01; ***p<0.0005; ****p<0.0001). These data support the conclusion that Poly(I:C) exposure disrupts short-term spatial working memory, with the combined exposure condition producing the most severe effect.

**Figure 3:**
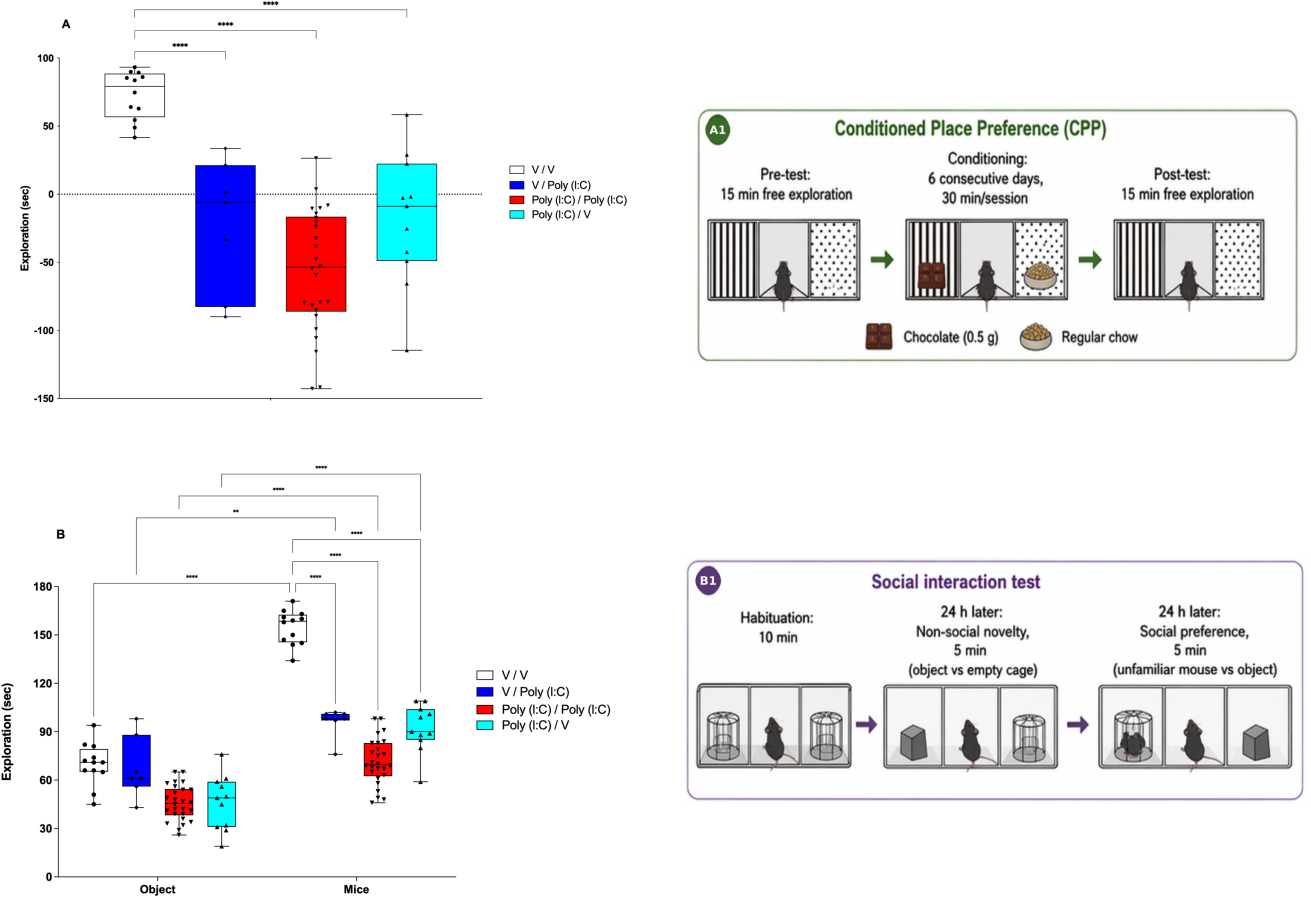
Performance in conditioned place preference (CPP) and social interaction test. Poly(I:C) exposure impairs reward-driven associative learning and attenuates social preference. **(A)** Chocolate-induced Conditioned Place Preference (CPP). After conditioning, control V/V mice showed positive exploration scores for the chocolate-paired compartment, indicating successful acquisition of place preference for palatable food-associated context, whereas Poly(I:C)-exposed groups (V/PolyI:C, PolyI:C/PolyI:C, PolyI:C/V) failed to develop CPP and displayed negative paired-compartment exploration scores (one-way ANOVA, F_(3,52)_ = 25.94, P<0,0001). (A1) Schematic illustration of the Conditioned Place Preference (CPP) behavioral paradigm. CPP consisted of pre-conditioning (establishing baseline preference), conditioning sessions by pairing one chamber with a stimulus (chocolate versus regular chow) and post-conditioning testing (measuring the change in preference). This drawing was created with BioRender.com. Data are presented as box-and-whisker plots with individual data points, and the horizontal line representing the median of the values. Data were analyzed by one-way ANOVA followed by Bonferroni’s *post hoc* test (**p<0.01; ***p<0.0005; ****p<0.0001*).* **(B)** Social interaction test: exploration of non-social (“Object”, left) versus social (unfamiliar mouse) stimuli across Poly(I:C) immune-challenge groups. Cumulative exploration time (sec) directed toward the object (left) or the unfamiliar conspecific (“Mice,” right) is shown for the four experimental groups: control V/V mice, V/PolyI:C, PolyI:C/PolyI:C/and PolyI:C/V. Overall, exploration was stimulus-dependent, with higher investigation of the social stimulus than the object, while exposure to Poly(I:C) altered the magnitude of social exploration among the groups (as indicated by multiple-comparison brackets) (two-way ANOVA, a significant main group effect F_(3,52)_ = 84,09, P<0,0001, a significant main stimulus effect F_(1,52)_ = 297.80, P<0,0001, and a significant group x stimulus effect F_(3,52)_ = 29.60, P<0,0001). (B1) Schematic illustration of the the social interaction behavioral paradigm. The social interaction task included habituation, non-social novelty exploration and social preference testing. This drawing was created with BioRender.com. Data are presented as box-and-whisker plots with individual data points, and the horizontal line representing the median of the values. Statistical analysis was performed using two-way ANOVA (factors: stimulus × group), Bonferroni’s *post hoc* test multiple comparisons (V/V N = 12; V/Poly(I:C) N = 7; Poly(I:C)/V N = 11; Poly(I:C)/Poly(I:C) N = 26; **p<0.01; ***p<0.0005; ****p<0.0001*)*.

To investigate reward-driven associative behavior, mice were subjected to a chocolate-induced conditioned place preference task. During the pre-conditioning phase, animals freely explored the apparatus to assess baseline chamber preference. As shown in **Figure 3A**, after conditioning, V/V mice displayed positive exploration scores for the chocolate-paired chamber, consistent with successful acquisition of a place preference for the palatable food-associated context. By contrast, V/PolyI:C, PolyI:C/V, and PolyI:C/PolyI:C mice did not show evidence of conditioned preference, as reflected by negative scores in the paired compartment rate (one-way ANOVA, Bonferroni’s *post-hoc* test; **p<0.01; ***p<0.0005; ****p<0.0001). These findings indicating that Poly(I:C) exposure impairs palatable food-induced CPP, with the strongest tendency toward impairment discernible in double-challenged PolyI:C/PolyI:C mice, further support the presence of alterations in reward-related and motivational processing.

Performance in the social preference task revealed a clear group-dependent alteration in social exploratory behavior. As shown in **Figure 3B**, control V/V mice exhibited the strongest preference for the social stimulus, as reflected by significantly greater exploration of the unfamiliar conspecific relative to the object. Although all groups maintained a preference for the social over the non-social stimulus, Poly(I:C)-exposed mice displayed a reduced magnitude of this response. All Poly(I:C)-treated mice showed lower levels of social investigation than V/V controls, in particular PolyI:C/PolyI:C animals that exhibited the greatest attenuation of social interaction rate (two-way ANOVA, Bonferroni’s *post-hoc* test; **p<0.01; ***p<0.0005; ****p<0.0001). Overall, these data indicate that Poly(I:C) exposure disrupts social investigation, with the most marked impairment occurring after double Poly(I:C) challenge.

### 3.2 Poly(I:C) immune challenge and impact on the amyloid precursor protein processing and tau phosphorylation

To assess whether repeated Poly(I:C) immune challenge affects molecular pathways commonly connected with AD-related neurodegeneration, including APP processing and tau phosphorylation (56), Western blotting analyses followed by semi-quantitative densitometry were carried out on total protein homogenates of hippocampus from animals of four experimental groups (V/V, V/PolyI:C, PolyI:C/V, and PolyI:C/PolyI:C). For probing, we used several commercial antibodies directed against Amyloid Precursor Protein (APP) -including 221C11 (66-81aa of N-terminus of APP) and anti-pan-C-terminal APP (751-770aa of C-terminus of APP) recognizing full-length APP and C-terminal soluble products (CTFs), respectively- beta-site APP-cleaving enzyme 1 (BACE1), Presenilin-1 (PSEN1), Neprilysin (NEP), full length tau protein and its AD-relevant phoshorylation sites (AT8, p^-^Tau-1). The hippocampus was chosen since: (i) this brain area is crucially involved in processing of episodic memory and early degenerates in conversion from Mild Cognitive Impairment (MCI) to Alzheimer disease (AD) (57); (ii) resident microglia is more immunologically active than in other brain regions (58, 59) and its activation precedes the synapses loss in the onset of AD (60).

**Figure 4:**
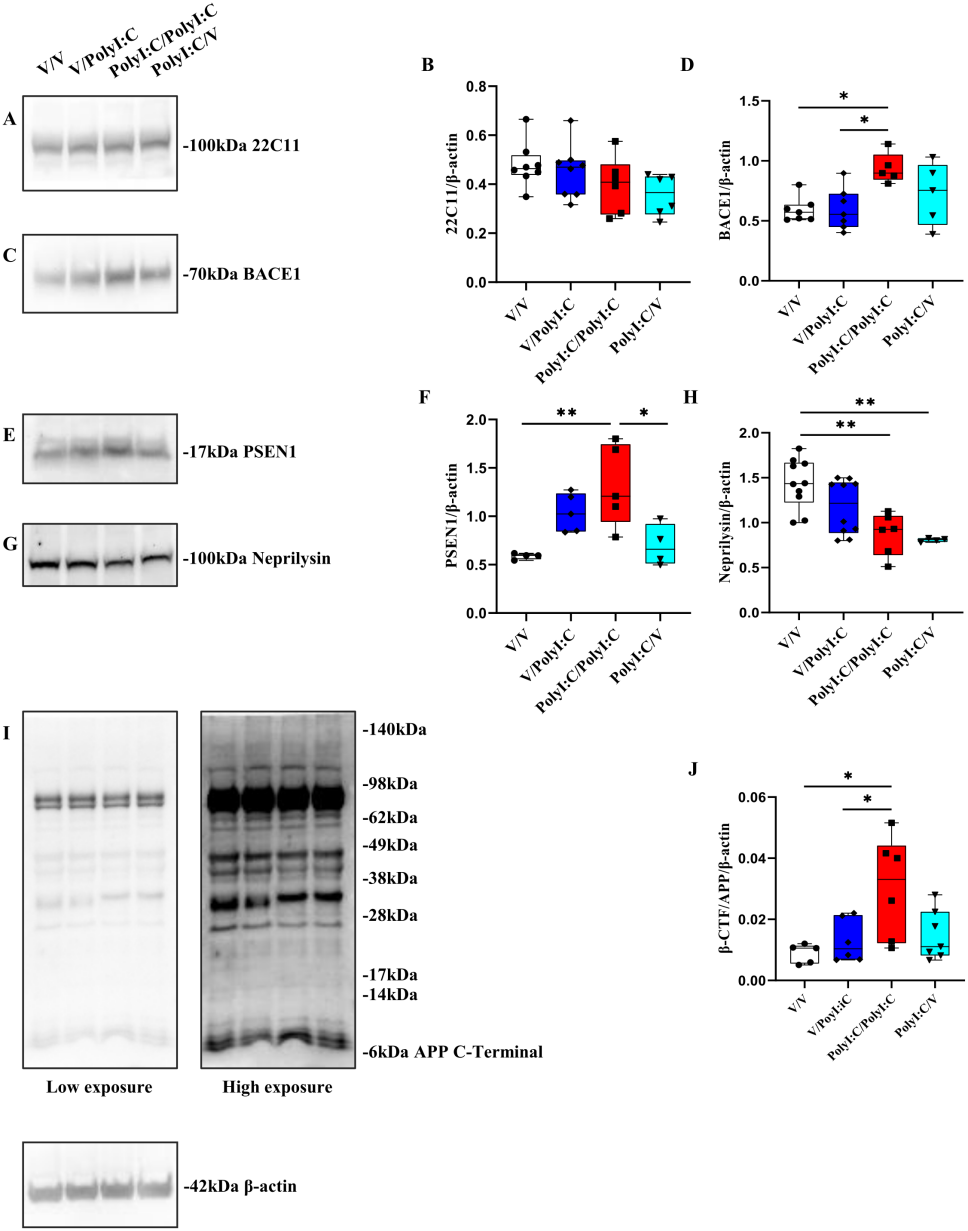
Amyloidogenic route of APP is activated in the hippocampus from PolyI:C/PolyI:C mice. **(A, C, E, G, I)** Representative images of Western blotting analysis (N = 4–10 animals per each group) carried out on whole protein lysates of hippocampi from animals of four experimental groups (V/V, V/PolyI:C, PolyI:C/PolyI:C and PolyI:C/V) with 22C11 (66-81aa of N-terminus of APP), BACE1, PSEN1, Neprilysin, anti-pan-C-terminal APP (751-770aa of C-terminus of APP) antibodies, as indicated alongside the blots. Arrows on the right side indicate the molecular weight (kDa) of bands calculated from migration of standard proteins. **(B, D, F, H, J)** Box-and-whisker plots show the semi-quantitative densitometry of the intensity signals in immunoreactivity bands by normalization with β-actin level used as loading control. β-actin band was from different gel of protein of interest. p<0.05 is accepted as statistically significant (one-way ANOVA, Bonferroni’s *post-hoc* test; **(B)** F_(3,24)_=2.071 p=0.1307; **(D)** F_(3,20)_=5.025 p=0.0093; **(F)** F_(3,14)_=6.837 p=0.0046; **(H)** F_(3,26)_=8.447 p=0.0004; **(J)** F_(3,20)_=4.999 p=0.0095; *p<0.05; **p<0.01.

**Figure 5:**
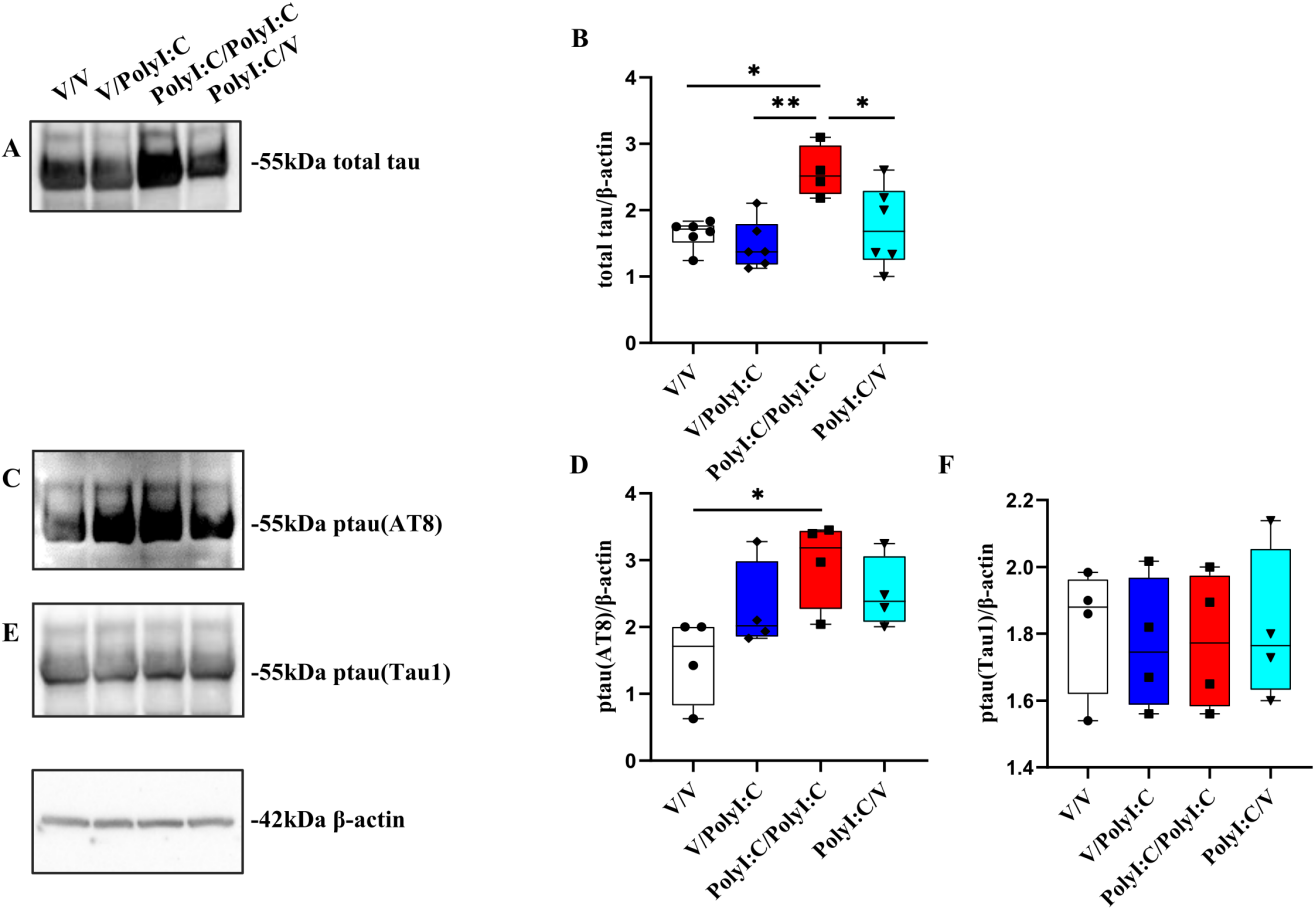
Site-specific hyperphosphorylation of tau is detected in the hippocampus from PolyI:C/PolyI:C mice. **(A, C, E)** Representative immunoblot (N = 4–6 animals per each group) of full length tau, AT8(p^+^Ser202/Thr205), Tau 1(p^-^Ser195/Ser198/Ser199/Ser202) in the hippocampal homogenates from animals of four experimental groups (V/V, V/PolyI:C, PolyI:C/PolyI:C and PolyI:C/V). **(B, D, F)** Box-and-whisker plots show the semi-quantitative densitometry of the intensity signals of bands by normalization with β-actin level used as loading control. β-actin band was from different gel of protein of interest. p<0.05 is accepted as statistically significant (one-way ANOVA, Bonferroni’s *post-hoc* test; **(B)** F_(3,18)_ =5.998 p=0.0051; **(D)** F_(3,12)_=3.718 p=0.0423; **(F)** F_(3,12)_=0.07043 p=0.9747; *p<0.05; **p<0.01; ****p<0.0001).

As shown in **Figures 4C–F**, into the hippocampus of double immune-challenged PolyI:C/PolyI:C experimental group, the endogenous steady-state expression levels of BACE1 and PSEN1, the β-secretase and the catalytic subunit of γ-secretase involved in amyloidogenic pathway of APP, respectively (61) were significantly increased (one-way ANOVA, Bonferroni’s *post-hoc* test; *p<0.05; **p<0.01). No change was detected in the band intensity of the full length APP precursor (**Figures 4A, B**) among the four experimental groups (one-way ANOVA, Bonferroni’s *post-hoc* test; p>0.9999). A significant up-regulation of C-terminal soluble fragment β (β-CTF or C99) (**Figures 4I, J**), which is a direct precursor of Amyloid beta (Aβ) released by BACE1 (62), together with a decline in NEP(**Figures 4G, H**), an Aβ-degradating endopeptidase (63), were also detected in PolyI:C/PolyI:C mice (one-way ANOVA, Bonferroni’s *post-hoc* test; *p<0.05; **p<0.01), suggesting an intracellular imbalance between its production and degradation as shown to occur in progression of AD (64). Interestingly, NEP expression level also appeared to be donwregulated in PolyI:C/V cohort (one-way ANOVA, Bonferroni’s *post-hoc* test; *p<0.05), in line with previous findings indicating that developmental immune challenge during late-gestional time window (GD17) is sufficient *per se* to predispose wild-type mouse offspring to AD-related brain aging features regardless the occurrence of a secondary aggravating inflammatory stimulus (48). Besides, we found out a marked increase in immunoreactivity level of full length tau (**Figures 5A, B**) (one-way ANOVA, Bonferroni’s *post-hoc* test; *p<0.05; **p<0.01) in PolyI:C/PolyI:C animals accompanied by an elevation in AT8 (p^+^Ser202/Thr205) phosphorylation site (one-way ANOVA, *post-hoc* test; *p<0.05) (**Figures 5C, D**), showing that repeated systemic inflammatory stimuli elevate the phosphorylation state of tau (65). No change was detected in immunoreactivity levels of non-phosho Tau-1 epitope (p^-^Ser195/Ser198/Ser199/Ser202)) (**Figures 5E, F**)among the four experimental groups (one-way ANOVA, Bonferroni’s *post-hoc* test; p>0.9999).

### 3.3 Poly(I:C) immune challenge and impact on microglia

Having established that prenatal immune activation and adult Poly(I:C) re-challenge are associated with APP and tau dysregulation, we next assessed the impact of double immune challenge with Poly(I:C) on neuroglia whose changes into pro-inflammatory states are tightly linked with AD pathophysiology by amplifying neuronal injury (66). To this aim, Western blotting analyses followed by semi-quantitative densitometry were carried out on total protein homogenates of hippocampus from animals of four cohorts (V/V, V/PolyI:C, PolyI:C/V, and PolyI:C/PolyI:C) probing with specific antibodies for Glial Fibrillary Acidic Protein (GFAP) and Ionized calcium-Binding Adaptor molecule 1 (Iba1), two well-established markers of astrocytosis and microgliosis respectively whose expression levels increase with their activated state (67, 68).

**Figure 6:**
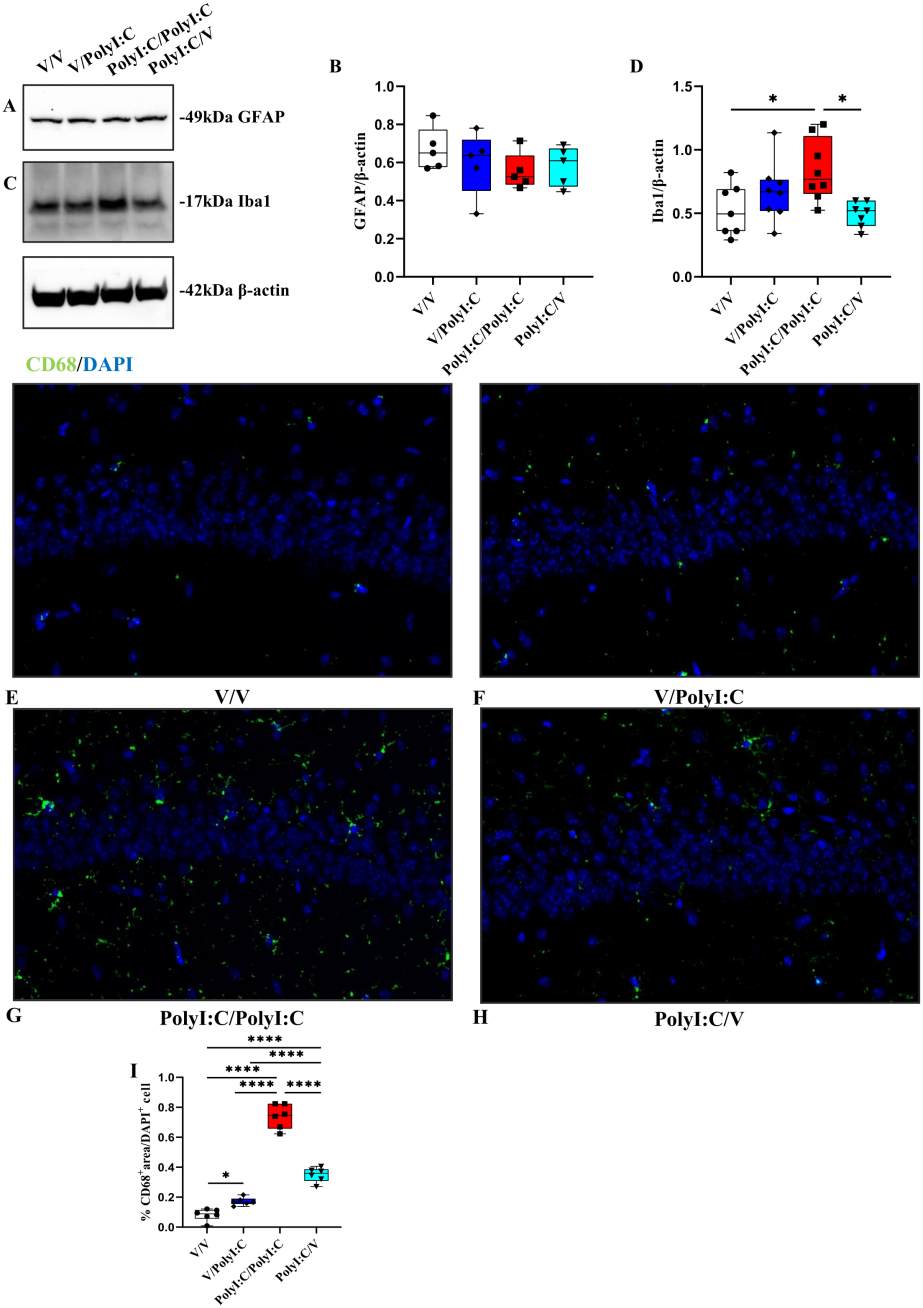
Systemic inflammation induces sustained microgliosis but does not affect astrocytes in the hippocampus from PolyI:C/PolyI:C mice. **(A, C)** Representative images of Western blotting analysis (N =5–8 animals per each group) carried out on whole protein lysates of hippocampi from animals of four experimental groups (V/V, V/PolyI:C, PolyI:C/PolyI:C and PolyI:C/V) with antibodies for GFAP, Iba1 (as indicated alongside the blots). Arrows on the right side indicate the molecular weight (kDa) of bands calculated from migration of standard proteins. **(B, D)** Box-and-whisker plots show the semi-quantitative densitometry of the intensity signals of bands by normalization with β-actin level used as loading control. β-actin band was from the same gel of protein of interest. p<0.05 is accepted as statistically significant (one-way ANOVA, Bonferroni’s *post-hoc* test; 6B: F_(3,16)_ =0.8215 p= 0.5009; 6D: F_(3,26)_=4.486 p=0.0115; *p<0.05; **p<0.01; ***p<0.0005; ****p<0.0001). **(E–H)** Microphotographers (20X) of CD68-labeled (green channel) microglial cells in hippocampal CA2 (cornu ammonis) region from coronal sections of animals (N = 6 animals per each group) of four experimental groups (V/V, V/PolyI:C, PolyI:C/PolyI:C and PolyI:C/V). Nuclei were counterstained by DAPI (blue channel) Scale bar= 20μm. **(I)** Fluorescence intensity quantification of the CD68 labeling in CA2 area from four experimental groups. p<0.05 is accepted as statistically significant (one-way ANOVA, Bonferroni’s *post-hoc* test; F_(3,20)_=181.3 p<0.0001; *p<0.05; ****p<0.0001).

As shown in **Figures 6C, D** the steady-state expression level of Iba1 was significantly increased in PolyI:C/PolyI:C animals (one-way ANOVA followed by Bonferroni’s *post-hoc* test; *p<0.05), in line with the notion that a prominent microglial activation is early visible in brains from AD specimens and animal models (69). On the contrary, no change in immunoreactivity level of GFAP (**Figures 6A, B**) was found among the four experimental groups (one-way ANOVA, Bonferroni’s *post-hoc* test; p>0.9999).

**Figure 7:**
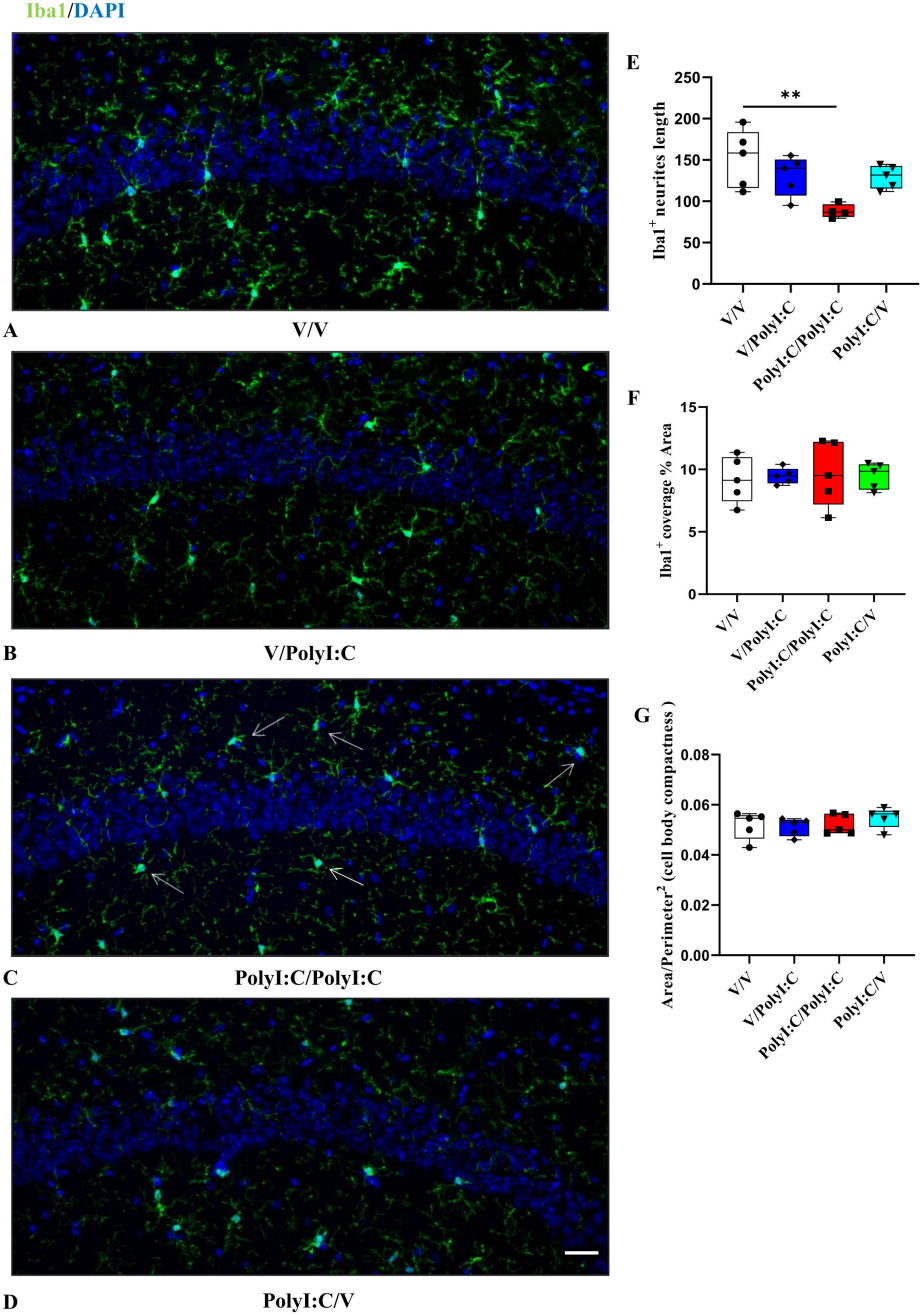
Hippocampal microglia from PolyI:C/PolyI:C mice undergoes morphological changes. **(A–D)** Representative microphotographers (N = 5 animals per each group) of hippocampal coronal sections (CA2 area) from animals of four experimental groups (V/V, V/PolyI:C, PolyI:C/PolyI:C and PolyI:C/V) stained for Iba1 (green channel) and DAPI (blue channel). Scale bar= 20μm. Arrows show dystrophic and de-ramified (reactive state) microglial morphology **(E–G)**. Quantification of neurites length **(E)**, coverage % area (Iba1-positive area/total ROI area×100) **(F)**, circularity (Area/Perimeter^2^) **(G)** of Iba1-positive microglial cells expressed in artibtrary unit (A.U.) p<0.05 is accepted as statistically significant (one-way ANOVA, Bonferroni’s *post-hoc* test; **(E)** F_(3,15)_=5.563 p=0.0091; **(F)** F_(3,16)_=0.06072 p=0.9797; **(G)** F_(3,16)_=0.6664 p=0.5848; **p<0.01.

Microgliosis was also confirmed by immunofluorescence analyses on coronal brain sections for expression of Cluster of Differentiation 68 (CD68), a lysosomal-associated membrane protein and marker for microglial phagocytic activity (70). In line with results from hippocampal-dependent tasks, a marked increase in punctate immunoreactivity in parenchyma of CA2 (**Figures 6E–I**), a key subfield critically involved in acquisition of both learning/memory and social behavior, was detected from PolyI:C/PolyI:C hippocampi (one-way ANOVA, Bonferroni’s *post-hoc* test; ****p<0.0001) when compared with the other three experimental groups, indicating that microglia acquired an activated phenotype. Consistent with this finding (**Figures 7A–D**), a morphological activation identified by a significant retraction of neuritic processes was also detectable in Iba1^+^-microglia from PolyI:C/PolyI:C mice (**Figure 7E**) (one-way ANOVA, Bonferroni’s *post-hoc* test; **p<0.01). No statistically significant change in coverage % Area (Iba1-positive area​/total ROI area×100) and compactness (area/perimeter^2^) of cell body (soma) (**Figures 7F, G** respectively) was detected among the four experimental groups (one-way ANOVA, *post-hoc* test; p>0.9999). A similar trend of microglial staining (CD68 and Iba 1 labeling) among the four experimental groups was detected throughout the different subfields (CA1, CA2, CA3, DG). Taken together, these findings indicate that under our experimental conditions activated, Iba1-overexpressing resident microglia adopts a dystrophic phenotype without significant enlargement in soma size and change in density or tissue occupancy, in agreement with their multifaceted, dynamic and gradual transition from resting ramified state toward reactive amoeboid profile (71, 72).

Next, we investigated whether a peripheral immune infiltration also took place into the animals’ brains, as previously reported to occur in several models of neuroinflammation (30) and in human AD (73). To this aim, immunofluorescence analyses were carried out on coronal hippocampal sections for expression of Cluster of Differentiation 45 (CD45), a receptor-linked protein tyrosine phosphatase that is expressed on all nucleated hematopoietic cells and is commonly used to distinguish resident microglia (CD45low) from infiltrating myeloid cells (CD45high) (74, 75). As shown (**Supplementary Figure 3**), an increased dot-like labeling was detected in V/PolyI:C, PolyI:C/V, and PolyI:C/PolyI:C experimental groups when compared to V/V controls (one-way ANOVA, Bonferroni’s *post-hoc* test; *p<0.05, ***p<0.0005) indicating the occurrence of infiltration of peripheral blood cells induced by systemic inflammation.

### 3.4 Poly(I:C) immune challenge and impact on synaptic and bioenergetics markers

After that we evaluated in this experimental animal model the expression of typical markers of synapses and energetic metabolism whose alterations in AD are early features preceding any histopathological and clinical manifestations (76, 77). To this aim, hippocampal lysates were analyzed by Western blotting with antibodies that recognize both pre- and post-synaptic proteins such as α-synuclein, Synaptosomal-Associated Protein 25kDa (SNAP25), syntaxin and PostSynaptic Density Protein 95 (PSD95). The levels of Neuronal nuclei (NeuN), a well-recognized transcriptional regulator used as marker of post-mitotic neurons (78) and of several both structural and functional mitochondrial proteins -including Voltage-Dependent Anion Channel (VDAC), Cytochrome c oxidase subunit IV (CoxIV), Translocase of Outer Mitochondrial Membrane 20 (Tom20), cytochrome C (CytC)- and of two critical enzymes for glycolytic flux, such Pyruvate Kinase (PKM2) and 6-Phosphofructo-2-Kinase/Fructose-2,6-Biphosphatase 3 (PFKB3), were also evaluated.

**Figure 8:**
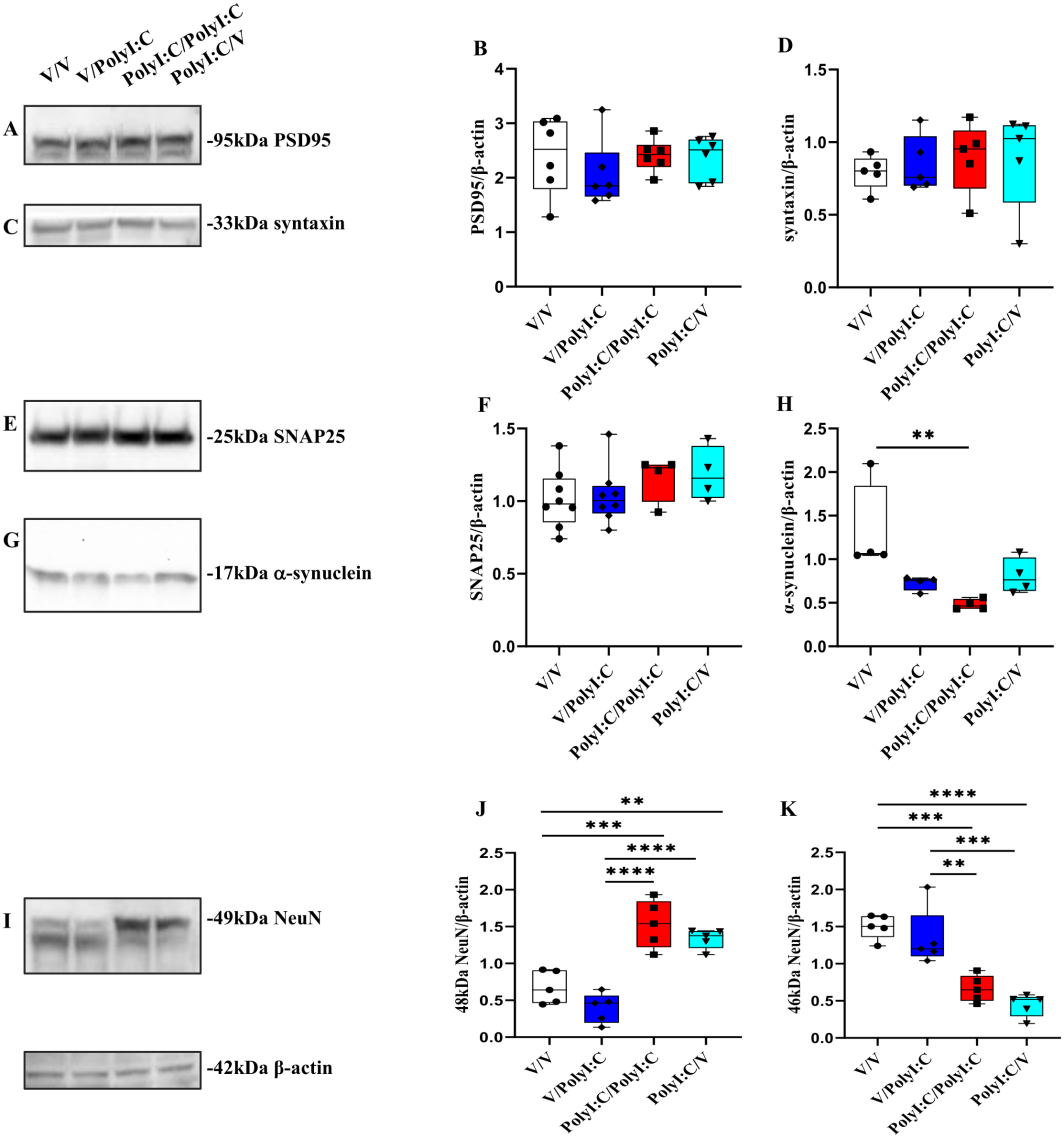
Loss of α-synuclein occurs in the hippocampus from PolyI:C/PolyI:C mice. **(A, C, E, G, I)** Representative immunoblot (N =4–8 animals per each group) of PSD95, syntaxin, SNAP25, α-synuclein, NeuN in the hippocampal homogenates from animals of four experimental groups (V/V, V/PolyI:C, PolyI:C/PolyI:C and PolyI:C/V). **(B, D, F, H, J, K)** Box-and-whisker plots show the semi-quantitative densitometry of the intensity signals of bands by normalization with β-actin level used as loading control. β-actin band was from different gel of protein of interest. p<0.05 is accepted as statistically significant (one-way ANOVA, Bonferroni’s *post-hoc* test; **(B)** F_(3,20)_=0.5718 p=0.6401; **(D)** F_(3,16)_=0.190 p=0.9015; **(H)** F_(3,12)_ =6.176 p=0.0088; **(J)** F_(3,16)_=27.01 p<0.0001; **(K)** F_(3,16)_ =22.11 p<0.0001; **p<0.01; ***p<0.0005; ****p<0.0001).

As shown in **Figures 8G, H**, the immunoreactivity level of α-synuclein, a presynaptic protein involved with Aβ and tau in pathophysiology of AD (79) whose expression is reduced both in human AD brains and animals models (80), was significantly decreased in PolyI:C/PolyI:C animals (one-way ANOVA, Bonferroni’s *post-hoc* test; **p<0.01). No change was contextually detected in the intensity bands of SNAP25 (**Figures 8E, F**), syntaxin (**Figures 8C, D**) and PSD95 (**Figures 8A, B**) among the four experimental groups (one-way ANOVA, Bonferroni’s *post-hoc* test; p>0.9999). Interestingly, NeuN signal quantitatively appears similar among the four cohorts suggesting that the Poly(I:C)- induced inflammation does not cause overt degeneration with change in survival of hippocampal neurons. Nevertheless, we found out an increment of the cytoplasmic 48kDa isoforms of NeuN -but not of the nuclear 46-kDa subtype- in PolyI:C/V and PolyI:C/PolyI:C groups when compared with V/V and V/PolyI:C (one-way ANOVA, Bonferroni’s *post-hoc* test; **p<0.01; ***p<0.0005; ****p<0.0001), providing an indication that systemic inflammation is more likely to cause defects in nucleocytoplasmic trafficking and changes in neuron-specific gene expression known to underly the molecular pathology of AD (81) rather than overall neuronal cell death.

**Figure 9:**
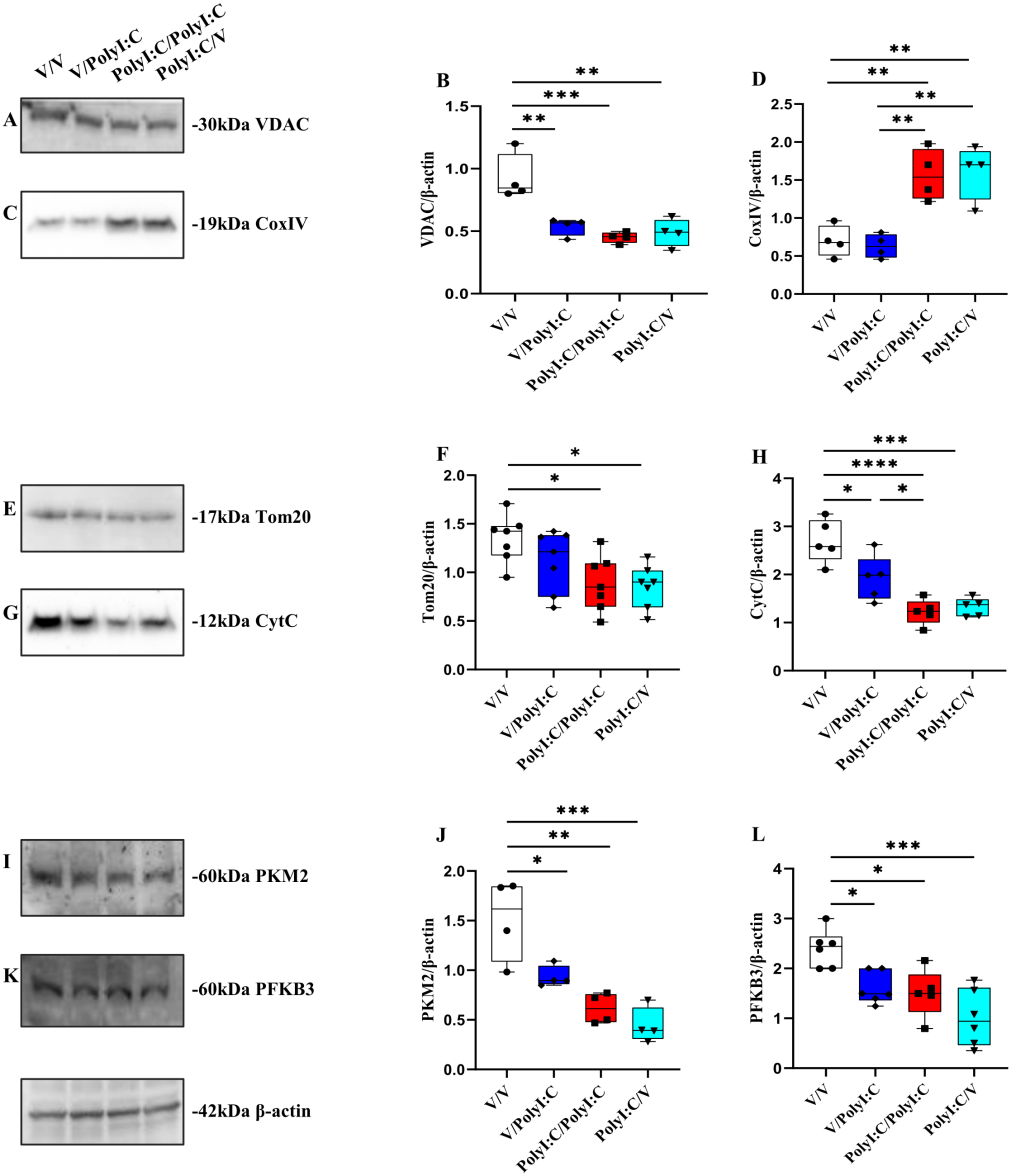
Mitochondrial and glycolitic markers are altered following immune challenge with Poly(I:C). **(A, C, E, G, I, K)** Representative immunoblot (N = 4–6 animals per each group) of VDAC, CoxIV, Tom20, CytC, PKM2 and PFKB3 in the hippocampal homogenates from animals of four experimental groups (V/V, V/PolyI:C, PolyI:C/PolyI:C and PolyI:C/V). **(B, D, F, H, J, L)** Box-and-whisker plots show the semi-quantitative densitometry of the intensity signals of bands by normalization with β-actin level used as loading control. β-actin band was from different gel of protein of interest. p<0.05 is accepted as statistically significant (one-way ANOVA, Bonferroni’s *post-hoc* test; **(B)** F_(3,12)_=14.11 p=0.0003; **(D)** F_(3,12)_:14.61 p=0.0003; **(F)** F_(3,24)_=5.095 p=0.0072; **(J)** F_(3,12)_=14.99 p=0.0002; **(L)** F_(3,19)_ =9.967 p=0.0004; *p<0.05; **p<0.01; ***p<0.0005.

Besides (**Figures 9A, B, E–L**), a decline in the band intensities of VDAC, Tom20, CytC, PKM2 and PFKB3 was visible both in PolyI:C/V and PolyI:C/PolyI:C animals (one-way ANOVA, Bonferroni’s *post-hoc* test; *p<0.05; **p<0.01; ***p<0.0005; ****p<0.0001) accompanied by an inverse, possibly compensatory, increase in CoxIV expression level (one-way ANOVA, Bonferroni’s *post-hoc* test; **p<0.01) (**Figures 9C, D**), indicating the occurrence of a more general, early-stage brain hypometabolism known to precede the bioenergetic collapse occurring in symptomatic AD (82). Interestingly, the expression levels of VDAC, CytC, PKM2 and PFKB3 turned out to be also reduced in V/PolyI:C cohort when compared with V/V controls (one-way ANOVA, Bonferroni’s *post-hoc* test; *p<0.05; **p<0.01), in line with the evidence that acute systemic inflammation regardless of the timing (prenatal and/or postnatal) has *per se* deleterious effect on brain bioenergetics (83).

## 4 Discussion

In the present study, we provide a mechanistic framework of how peripheral immune activation negatively influences brain physiology and behaviour in PolyI:C/PolyI:C animals that represent a unique tool to investigate the molecular mechanisms of neuroinflammatory and neurodegenerative processes without interference due to transgene overexpression (41). In particular, we show that double immune challenge following systemic administration of the viral mimetic Poly(I:C) (i.e. exposure of non-transgenic mouse dams to Poly(I:C) in late-gestional stage followed by secondary immune challenge in offspring at 9 months of age) has in adulthood (12 months) long-term deleterious effects on cognitive and motivational performances that are paralleled by the occurrence of AD-related molecular and cellular alterations, including aberrant amyloidogenic APP processing with production of β-CTF fragment, site-specific hyperphosphorylation of tau at p^+^Ser202/Thr205 (AT8) residues, microgliosis with increased phagocytic activity and process retraction, synaptic loss (α-synuclein) and bioenergetic deficits.

At the behavioral level, Poly(I:C)-exposed mice exhibited impairments across multiple domains. In cognitive tasks, both recognition memory (novel object recognition test) and short-term spatial working memory (Y-maze spontaneous alternation) were significantly reduced, indicating deficits in hippocampus-dependent memory functions. In addition to cognitive alterations, non-cognitive behavioral domains were also affected under our experimental conditions. In the CPP task, Poly(I:C)-treated animals failed to develop a preference for the palatable food-associated context, suggesting alterations in reward-related and motivational processing. A reduction in social investigation was also detected, reflecting altered responsiveness to social stimuli. In particular, these behavioral impairments were most pronounced in double-challenged PolyI:C/PolyI:C animals, supporting the idea that repeated immune activation exacerbates functional outcomes both of cognitive and non-cognitive domains. To this regard, it is important to underline that the detected impairments in reward-related and social behaviors should be interpreted with caution since they may reflect broader alterations in motivational or affective states rather than domain-specific deficits. Nevertheless, whereas sickness-like responses are classically described as acute consequences of systemic immune activation, the long-term interval imposed by our experimental design between the second immune challenge (9M) and the behavioral assessment (12M) further strengthen the occurrence of persistent alterations in behavioral regulation in Poly(I:C)-treated animals. Accordingly, these behavioral alterations are interpreted in conjunction with the accompanying molecular and cellular changes, rather than as standalone indicators of AD-related dysfunction. Importantly, the deficits we found out in recognition memory and spatial working memory are consistent with the well-established role of the hippocampus in these cognitive domains (84). Although reward-related and social behaviors are not exclusively mediated by the hippocampus, this brain region contributes to the integration of contextual, motivational, and social information through its interactions with distributed cortico-limbic circuits (85, 86).Moreover, an interesting study have reported that the immune stimulation with Poly(I:C) at GD17 does not impair in adulthood the sensorimotor gating assessed using the paradigm of PrePulse Inhibition (PPI) of the acoustic startle response but working memory, offering thus insights into its potential underlying mechanisms (87).

Notably, and in line with previous findings using the same timing and dosing of Poly(I:C) (48), we demonstrate that the deficits in cognitive, motivational and social behavior along with signs of neuronal degeneration and microglia activation manifest in the hippocampus of double immune-challenged PolyI:C/PolyI:C mice at greater extent than in other three experimental groups, indicating that sustained peripheral immune stimulation by repeated exposure to Poly(I:C) induces long-lasting neuroimmune priming/reprogramming that is sufficient to elicit a constellation of molecular, cellular and behavioral changes overlapping with features relevant to AD-associated neurodegenerative vulnerability. The convergence of behavioral impairment with AD-relevant biochemical and morphological alterations we found out in PolyI:C/PolyI:C animals fosters an immunology-centered model of processes relevant to sporadic AD, rather than recapitulating the full-blown staging of human plaque/tangle neuropathology. Taken together our behavioural, morphological and biochemical results support the notion that neuroimmune derangement is not merely a secondary response (4, 5) but that the dysfunction of brain’s immune system following peripheral immune stimulation with inflammatory insult may represent a contributing factor in the development of neurodegenerative processes associated with the most common, sporadic form of AD. Furthermore, we found out higher level of pro-inflammatory TNF-α both in plasmatic and hippocampal samples from Poly(I:C)-challenged mice (V/PolyI:C) in comparison with vehicle controls (V/V) (**Supplementary Figure 4**), thus supporting the occurrence of a primed state, in which monocytes circulating in the peripheral blood or CNS resident microglia show increased inflammatory responses. Elucidating the mechanisms that govern the cross-talk between peripheral-central immune systems helps to reconsider neurodegeneration as a multi-organ process and to advance the development of inflammation-targeted therapeutic avenues that are effective in changing the AD clinical trajectory in its initial and, then, modifiable phases (88, 89).

In support of the pathophysiological link between priming of innate immunity and AD etiology, previous studies have shown that chronic systemic inflammation is risk factor for cognitive impairment in rodent models (90–92) and in humans (93–97). Consistently, peripheral immune stimulation with LPS affects in old wild type mice the neuronal morphology of CA1 pyramidal neurons and Dentate Gyrus (DG) granule cells (dendritic complexity and spine density) and causes impairment in Long-Term Potentiation (LTP) and spatial learning in Morris water maze navigation task, depending on microglial NOD-, LRR- and pyrin domain-containing protein 3 (NLRP3) inflammosome activation (98). Interestingly, Aβ itself can act as Damage-Associated Molecular Pattern(s) (DAMPs) and “primes” microglia upon binding to different receptors, including TLRs, followed by activation of the NLRP3 inflammosome (99). This primed phenotype renders microglia more sensitive to peripheral challenges creating a persisting brain inflammatory milieu that not only reduces the ability of microglia to clear Aβ plaques with consequent accumulation in the parenchyma but also damages neuronal circuits (100). This interpretation aligns with the evidence that systemic inflammation caused by LPS inoculation damages the microglial Aβ clearance via NLRP inflammosome aggravating the cognitive deficits in APP/PS1 mice (30). Hippocampal microglia from sepsis-surviving mice shows an increased susceptibility when exposed to low amounts of Aβ oligomers (AβO) shifting towards an activated morphology with enhanced secretion of several pro-inflammatory cytokines (IL-1β, IL-6, INF-γ) and its *in vivo* depletion by means of treatment with colony stimulating factor 1 receptor inhibitor (PLX3397) significantly prevents cognitive impaiment induced by exposure of animals to AβO (101), further strenghening the evidence that systemic inflammation is a modifier of AD neurodegeneration. In this context, Poly(I:C) provides a complementary viral-mimetic trigger that engages innate immune programs relevant to infection-related inflammatory episodes, in line with the finding that the stronger phenotype we found out in the double-hit group PolyI:C/PolyI:C mimics a priming/re-challenge paradigm rather than a transient acute inflammatory effect (41, 48). In a prospective study, acute systemic inflammation causes a two-fold increase in the rate of cognitive decline in subjects with mild AD with a positive correlation between serum levels of TNF-α and memory dysfunction (102). Genetic risk factor (APOE, TREM-2) combined with environmental inflammatory conditions (diet, aging, vascular damage, infection, microbial dysbiosis, accumulation of endogenous misfolded proteins of Aβ and tau) increase the progression to a more severe dementia in AD subjects (103). Interestingly, in a study carried out on 22 twins pairs, five twins with systemic infection developed AD at an earlier age than their corresponding co-twin (104). This scenario fits well with what we describe in this experimental animal model, where repeated systemic inflammatory challenges mimicking viral invasion shape AD-related molecular, cellular and behavioral alterations.

**Figure 10:**
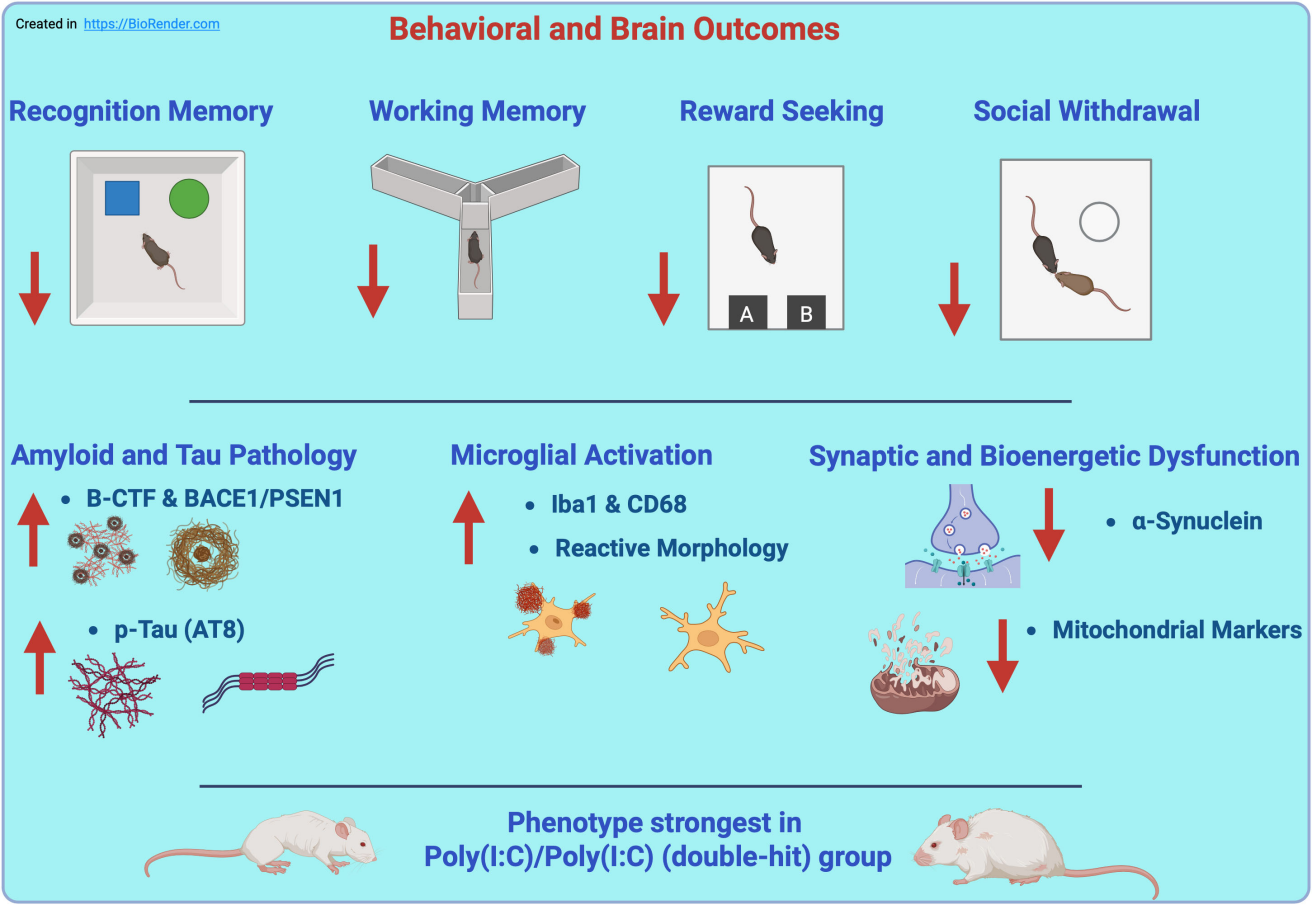
Schematic summary of the convergent behavioural and neuropathological outcomes detected in double-hit Poly(I:C) paradigm. Impaired recognition and working memory, reduced reward-seeking behavior in the conditioned place preference task and social investigation, enhanced amyloidogenic APP processing (β-CTF accumulation with increased BACE1/PSEN1) in the hippocampus along with increased tau phosphorylation at the AT8 epitope, microglial activation (increased Iba1 and CD68 immunoreactivity with reactive morphological remodeling) and synaptic/bioenergetic dysfunction (reduced α-synuclein and reductions in mitochondrial and glycolytic markers) were all detected in wild-type mice following Poly(I:C) immune challenge. Notably, the most robust behavioral and hippocampal molecular alterations were observed in the double-hit PolyI:C/PolyI:C group. This drawing was created with BioRender.com.

Another key finding of this study is that astrocytes, which are also able to communicate with immune system (105, 106) and promotes neuroinflammation in AD (107) do not show any significant alterations, in agreement with previous studies showing that microglia is the first immune-competent brain cell sensing DAMP signals from the peripheral inflammation (108). Since reactivity of astrocytes can be heterogeneous just like that of microglia (36), future studies using multiple astrocytic markers beyond the GFAP immunoreactivy and/or transcriptomic profiling will be required to rule out the involvement, possibly at later staging, of any change in their functional state. To this point, we found out that hippocampal microglial cells from PolyI:C/PolyI:C mice appear to be “primed” since they express high levels of Iba-1 and lysosomal protein CD68 markers and undergo changes in morphology with de-ramification, both indicative of their functional hyperactivation. This finding reinforces the occurrence of long-lasting neuroimmune priming mechanism in the “double-hit” Poly(I:C) paradigm. However, future studies aimed at assessing the post-MIA return-to-baseline trajectories and durable myeloid reprogramming (e.g., enhanced cytokine output upon re-stimulation together with metabolic/epigenetic signatures) following the second challenge are still required to distinguish persistent low-grade inflammation from trained immunity mechanism(s) (109). Besides, the evidence that the immune priming is largely mediated by brain-resident macrophages (microglia), but not by astrocytes, with a moderate infiltration of circulating leukocytes upholds a model in which peripheral innate immune activation propagates to the brain through peripheral-to-central immune communication rather than direct Poly(I:C) entry into the parenchyma. Consistently, microglia and monocytes both in the brain and in the periphery are currently targeted both in pre-clinical and clinical studies for the development of new therapeutic strategies aimed at mitigating cognitive decline and improving mood in AD settings (89). A graphical summary of the “double-hit” paradigm and the convergent behavioral and molecular outcomes detected in Poly(I:C)-challenged mice is provided in **Figure 10**.

From a mechanistic point of view, a large body of evidence shows that the peripheral activation of innate immune system upon repeated exposure to inflammation-inducing agents initiates a pro-inflammatory signaling cascade that is transmitted/propagated to and further amplified in the brain parenchyma by resident, sensitized or “primed” microglia (110). Direct diffusion via the circumventricular organs lacking of Blood Brain Barrier (BBB), indirect engagement of Pattern-Recognition Receptors (PRRs) on brain endothelial cells and perivascular macrophages and neuronal propagation along the vagus nerve are all possible routes of this peripheral-central neuroimmune communication (111, 112). The priming of microglia and their hyperactivation under pro-inflammatory conditions is considered to be a maladaptive unresolved response to the initial/recurrent stimulus and is accompanied by alterations in morphology, increased proliferation and upregulation in expression of cell surfaces receptors and intracellular molecules (19). Driven by prolonged and exaggerated release of cytokines and other pro-inflammatory mediators, this phenomenon is likely to trigger a vicious cycle at the interplay neuron-glia leading eventually to synaptic damage, pathological protein aggregation, and impaired clearance, cytoskeleton abnormalities, energetic imbalance and neuronal injury, psychiatric symptoms and cognitive deficits (27). Consistent with the existence of a neuroimmune pathway that traffics the inflammatory signals from the periphery to brain, microglia is not directly exposed to circulating Poly(I:C) that causes a systemic increase in the production of pro-inflammatory cytokines upon binding to Toll-like receptor (TLR) 3 expressed on the surface of peripheral macrophages and dendritic cells without crossing the BBB and reaching the brain parenchyma (41). In the specific context of Poly(I:C), peripheral TLR3-driven inflammatory programs (including interferon-linked signaling) may provide a sustained systemic input to the neurovascular unit and CNS interfaces, thereby promoting microglial priming and facilitating leukocyte recruitment, even in the absence of direct parenchymal exposure to Poly(I:C).

Limitations of the study should be acknowledged. First, only male offspring were analyzed, and this limits the generalizability of the findings, as sex-dependent differences in innate immune responses and neurodegenerative processes (e.g., microglial reactivity) may significantly influence vulnerability to neuroimmune priming and associated alterations. Future studies including female cohorts will be necessary to determine the extent to which these results can be expanded to both sexes. Second, the present work focuses on hippocampal molecular and cellular readouts. Future studies integrating the peripheral cytokine kinetics, myeloid cell phenotyping and region-wide brain mapping (including cortex) will better define the systemic-to-central immune trajectory. Third, we did not quantify Aβ42/Aβ40 levels, soluble Aβ oligomers, or amyloid plaque burden. Therefore, although the APP processing and tau phosphorylation signatures are consistent with an AD-like neuropathology, the present data do not recapitulate a full-blown AD neuropathology but rather, indicate that repeated Poly(I:C) immune challenge promotes AD-like alterations in APP and tau metabolism, together with microglial, synaptic, bioenergetic and behavioral abnormalities. Fourth, increased CD45 immunoreactivity is consistent with peripheral immune involvement but tissue-based CD45 signal alone cannot fully resolve cell identity and compartment (e.g., border-associated immune cells versus parenchymal infiltration). Complementary approaches such as flow cytometry or single-cell profiling will strengthen the inference. Fifth, the phenotype was assessed at a single aging time point (12 months) and longitudinal sampling will be needed to better define the temporal sequence between peripheral immune activation, neuroimmune priming and downstream APP/tau, synaptic, and metabolic changes. Finally, the present findings should be interpreted within the boundaries of their experimental design. Although the described molecular and behavioral alterations resemble the main features of sporadic AD, this model does not aim at recapitulating the full spectrum of AD pathology but rather at phenocopying in wild-type mice the innate immunity-related neurodegenerative changes with the intent of providing a framework to investigate inflammation-dependent vulnerability pathways.

## 5 Conclusion

Sustained activation of peripheral innate immunity by repeated exposure to Poly(I:C) was sufficient in wild-type mice to elicit long-lasting, cognitive and non-cognitive dysfunction along with hippocampal molecular and cellular changes relevant to AD-associated neurodegenerative vulnerability. These findings support an immunology-centric model in which innate immune priming associated with microglial hyperactivation and peripheral immune infiltration may contribute to downstream alterations including amyloidogenic processing, tau dysregulation, synaptic vulnerability and bioenergetic impairment. A key mechanistic implication is that systemic stimulation of innate immunity can be biologically “transduced” to the brain through peripheral-to-central neuroimmune communication. Thus, inflammatory mediators released in the periphery (e.g., cytokines/chemokines and type I interferon programs) may activate CNS interface tissues (meninges, choroid plexus, perivascular spaces) and promote trafficking of peripheral myeloid cells into brain border compartments and/or parenchyma, thereby driving microglial priming and sustaining neuroinflammatory signaling. In this framework, therapeutic leverage points include both maladaptive innate immune mechanisms in peripheral myeloid compartments and the systemic-to-brain inflammatory relay at neurovascular and CNS interface sites which together may shape vulnerability trajectories relevant to sporadic AD. Targeting maladaptive innate immunity and the systemic-to-brain inflammatory relay at these interfaces may therefore represent a valuable strategy to potentially influence vulnerability trajectories relevant to sporadic AD.

## Data Availability

The raw data supporting the conclusions of this article will be made available by the authors, without undue reservation.

## Supplementary material

Supplementary Figure 1 Negative controls for Western blotting and immunofluorescence. **(A, B)** The absence of signal following the omission of the primary antibody was used as negative control for nonspecific binding of the secondary antibody in Western Blotting analyses and Immunofluorescences on homogenates and coronal sections of hippocampus, respectively **(D, E)**. Nuclei were stained with DAPI. Ponceau staining **(C)** is also shown to visualize the protein ladder. Scale bar= 20μm.

Supplementary Figure 2 Locomotor activity was unchanged among the four experimental groups. Distance travelled by animals of four experimental groups (V/V, V/PolyI:C, PolyI:C/PolyI:C and Poly(I:C)/V) during the habituation session (one-way ANOVA, Bonferroni’s *post-hoc* test; F_(3,52)_=0.3, p=0.77). p<0.05 accepted as statistically significant.

Supplementary Figure 3 Peripheral immune infiltration is visible in the hippocampus of wild-type mice regardless of the timing (pre- versus postnatal) and the dosing (single versus double challenge) of PolyI:C infusion. **(A-D)** Representative (N = 6 animals per each group) microphotographers of hippocampal coronal sections (Dental Gyrus area) from animals of four experimental groups (V/V, V/PolyI:C, PolyI:C/PolyI:C and PolyI:C/V) stained for CD45 (green channel) and DAPI (blue channel). Scale bar= 20μm. **(E)** Bar graph shows the fluorescence intensity quantification of the CD45 labeling from four experimental groups. p<0.05 is accepted as statistically significant (one-way ANOVA, Bonferroni’s *post-hoc* test; F_(3,20)_=12.44 p<0.0001; *p<0.05; **p<0.01; ***p<0.0005; ****p<0.0001).

Supplementary Figure 4 TNF-α levels are elevated both in plasma and hippocampus of V/PolyI: Cmice in comparison to V/V controls. ELISA assays on plasma following **(A)** *in vitro* whole blood stimulation with 2.5 µg/mL of R848, a dual agonist for Toll-like receptors 7 and 8 (unpaired t-test, t=7.32, df=2, p=0.018), and **(B)** on hippocampal homogenates of V/PolyI:C and V/V mice (unpaired t-test, t=5,64, df=2, p=0.03, p<0.05 accepted as statistically significant.

Supplementary Figure 5 Uncropped images of Western blotting analyses of **Figure 5**.

Supplementary Figure 6 Uncropped images of Western blotting analyses of **Figure 6**.

Supplementary Figure 7 Uncropped images of Western blotting analyses of **Figure 7**.

Supplementary Figure 8 Uncropped images of Western blotting analyses of **Figure 9**.

Supplementary Figure 9 Uncropped images of Western blotting analyses of **Figure 10**.
