# Supplementary figures and images for: Prenatal immune activation and adult Poly(I:C) re-challenge promote neuroimmune priming and AD-related behavioural, cellular and molecular alterations in wild-type mice
Source: Front Immunol. 2026 Jul 8;17:1845312. doi: 10.3389/fimmu.2026.1845312 (PMC13403630; doi:10.3389/fimmu.2026.1845312)

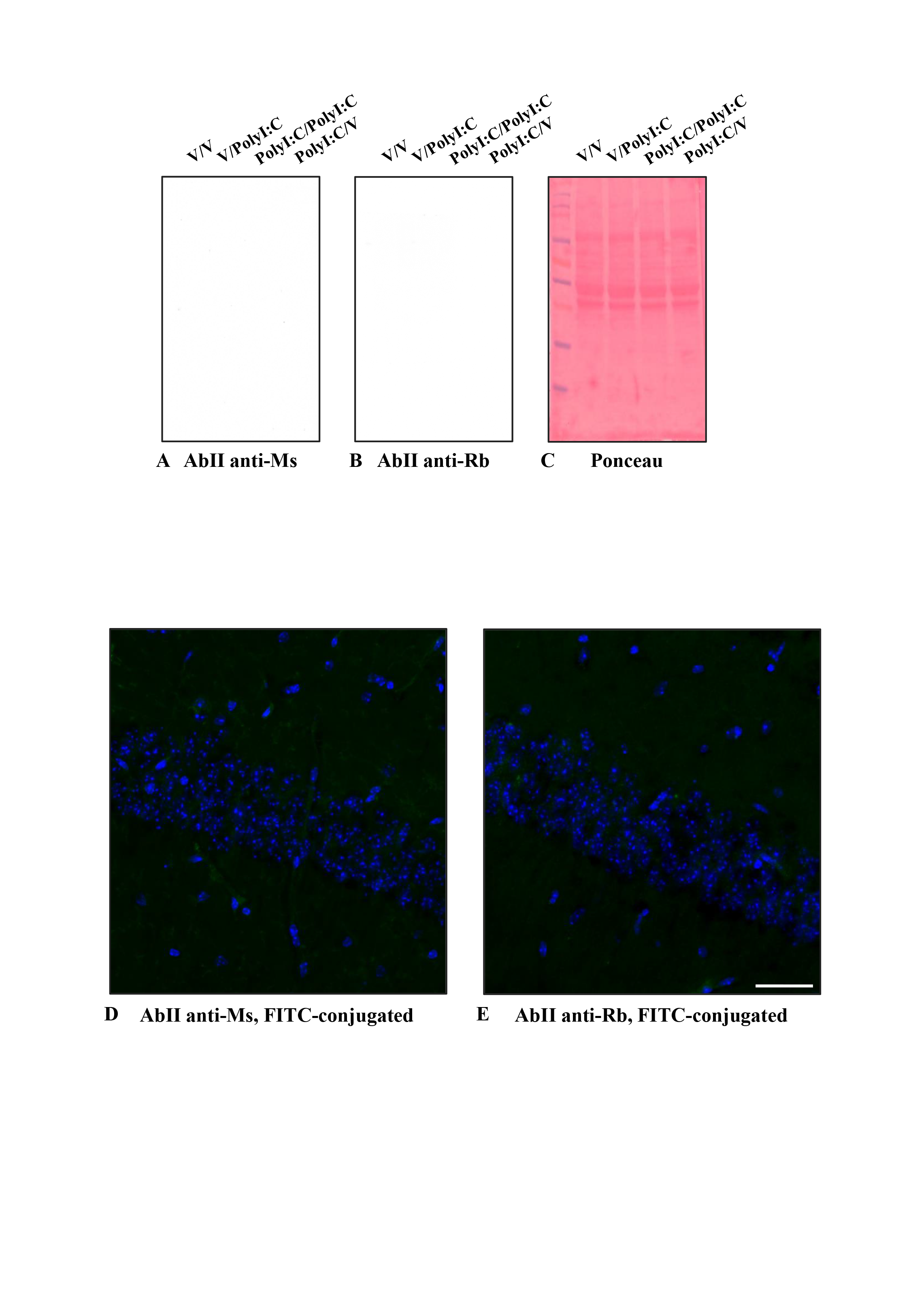

Supplement: Supplementary Figure 1 — Negative controls for Western blotting and immunofluorescence. (A, B) The absence of signal following the omission of the primary antibody was used as negative control for nonspecific binding of the secondary antibody in Western Blotting analyses and Immunofluorescences on homogenates and coronal sections of hippocampus, respectively (D, E). Nuclei were stained with DAPI. Ponceau staining (C) is also shown to visualize the protein ladder. Scale bar= 20μm. [file Image1.tif]

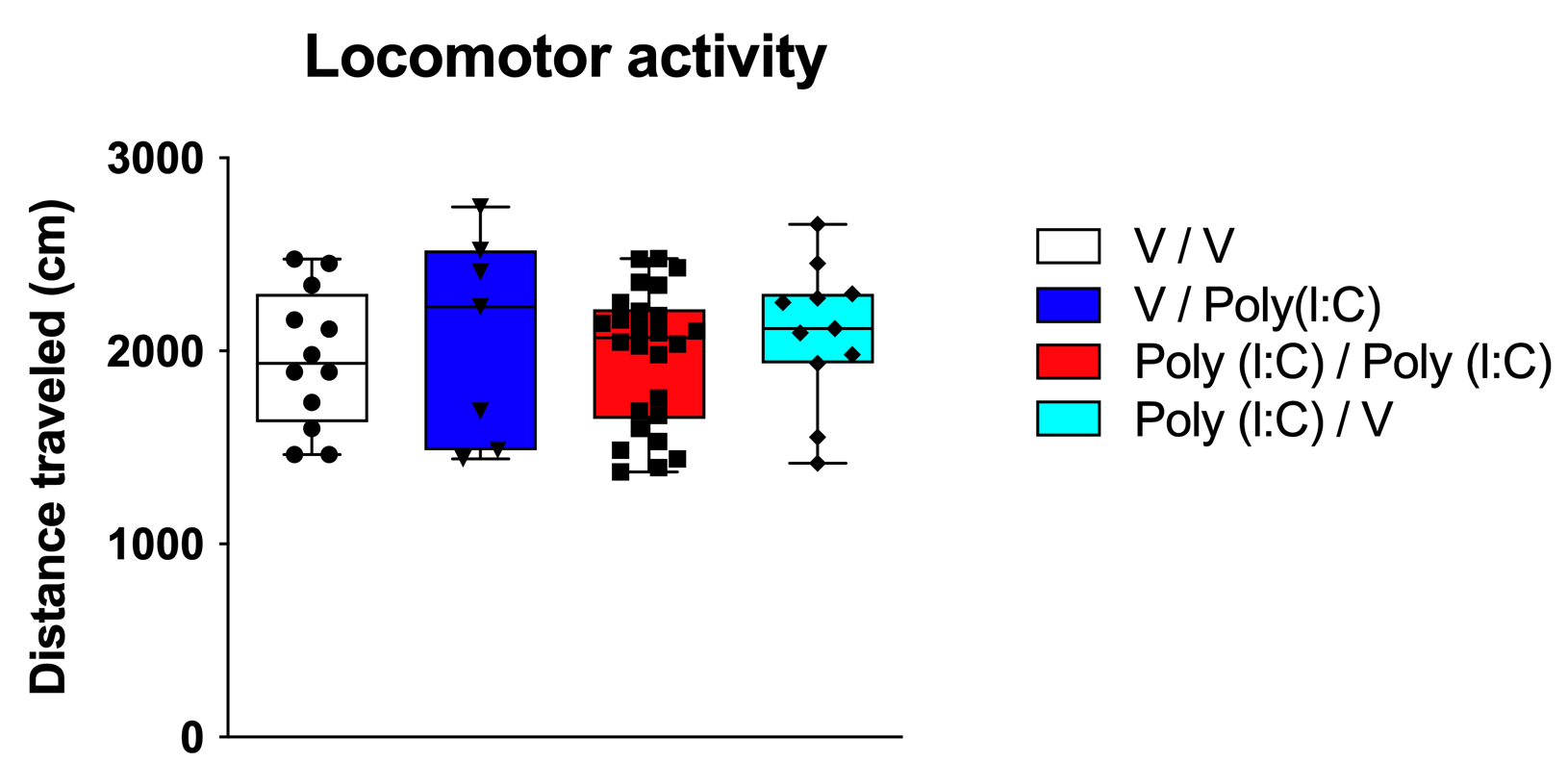

Supplement: Supplementary Figure 2 — Locomotor activity was unchanged among the four experimental groups. Distance travelled by animals of four experimental groups (V/V, V/PolyI:C, PolyI:C/PolyI:C and Poly(I:C)/V) during the habituation session (one-way ANOVA, Bonferroni’s post-hoc test; F(3,52)=0.3, p=0.77). p<0.05 accepted as statistically significant. [file Image2.tiff]

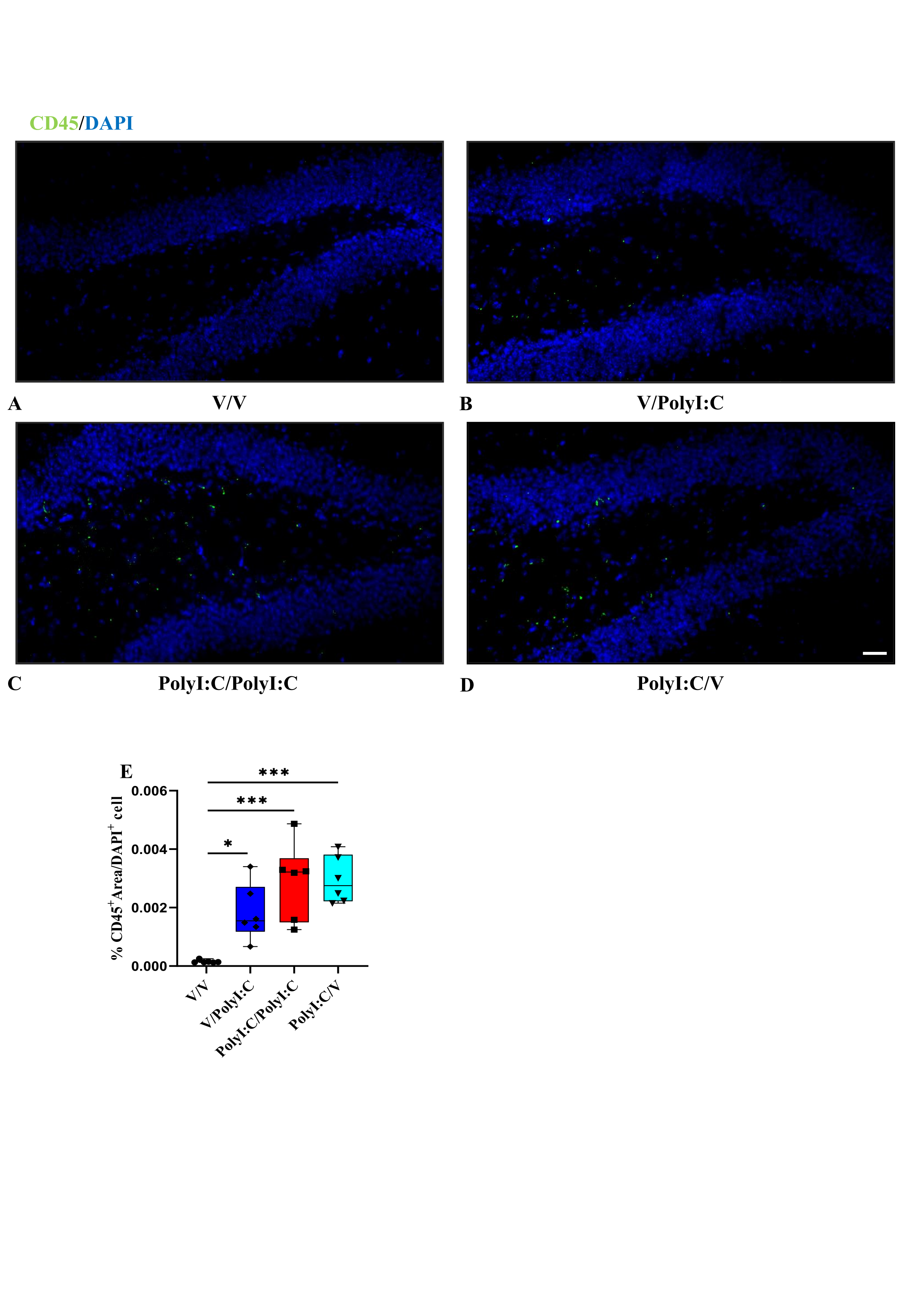

Supplement: Supplementary Figure 3 — Peripheral immune infiltration is visible in the hippocampus of wild-type mice regardless of the timing (pre- versus postnatal) and the dosing (single versus double challenge) of PolyI:C infusion. (A-D) Representative (N = 6 animals per each group) microphotographers of hippocampal coronal sections (Dental Gyrus area) from animals of four experimental groups (V/V, V/PolyI:C, PolyI:C/PolyI:C and PolyI:C/V) stained for CD45 (green channel) and DAPI (blue channel). Scale bar= 20μm. (E) Bar graph shows the fluorescence intensity quantification of the CD45 labeling from four experimental groups. p<0.05 is accepted as statistically significant (one-way ANOVA, Bonferroni’s post-hoc test; F(3,20)=12.44 p<0.0001; *p<0.05; **p<0.01; ***p<0.0005; ****p<0.0001). [file Image3.tif]

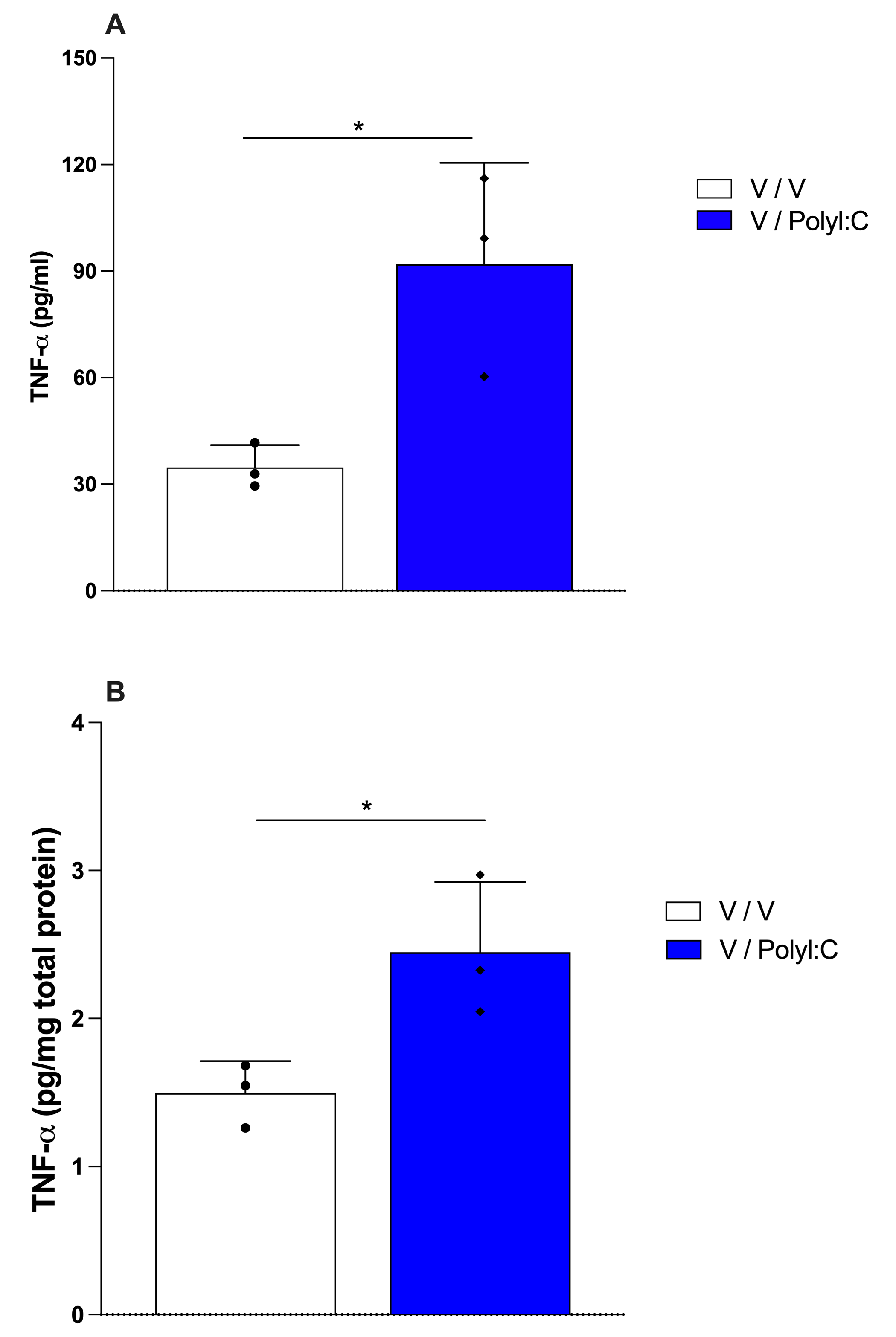

Supplement: Supplementary Figure 4 — TNF-α levels are elevated both in plasma and hippocampus of V/PolyI: Cmice in comparison to V/V controls. ELISA assays on plasma following (A) in vitro whole blood stimulation with 2.5 µg/mL of R848, a dual agonist for Toll-like receptors 7 and 8 (unpaired t-test, t=7.32, df=2, p=0.018), and (B) on hippocampal homogenates of V/PolyI:C and V/V mice (unpaired t-test, t=5,64, df=2, p=0.03, p<0.05 accepted as statistically significant. [file Image4.tiff]

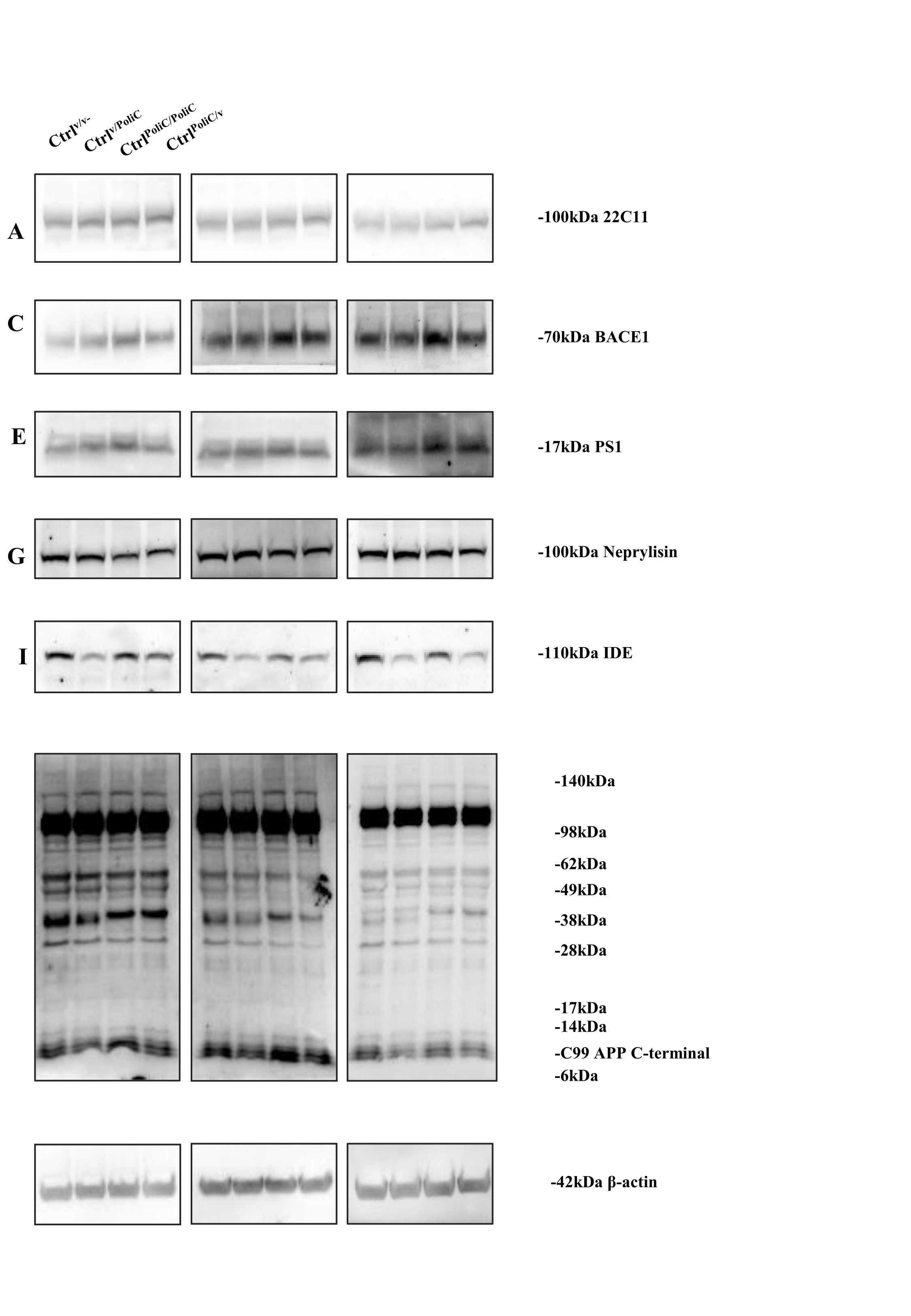

Supplement: Supplementary Figure 5 — Uncropped images of Western blotting analyses of Figure 5. [file Image5.tif]

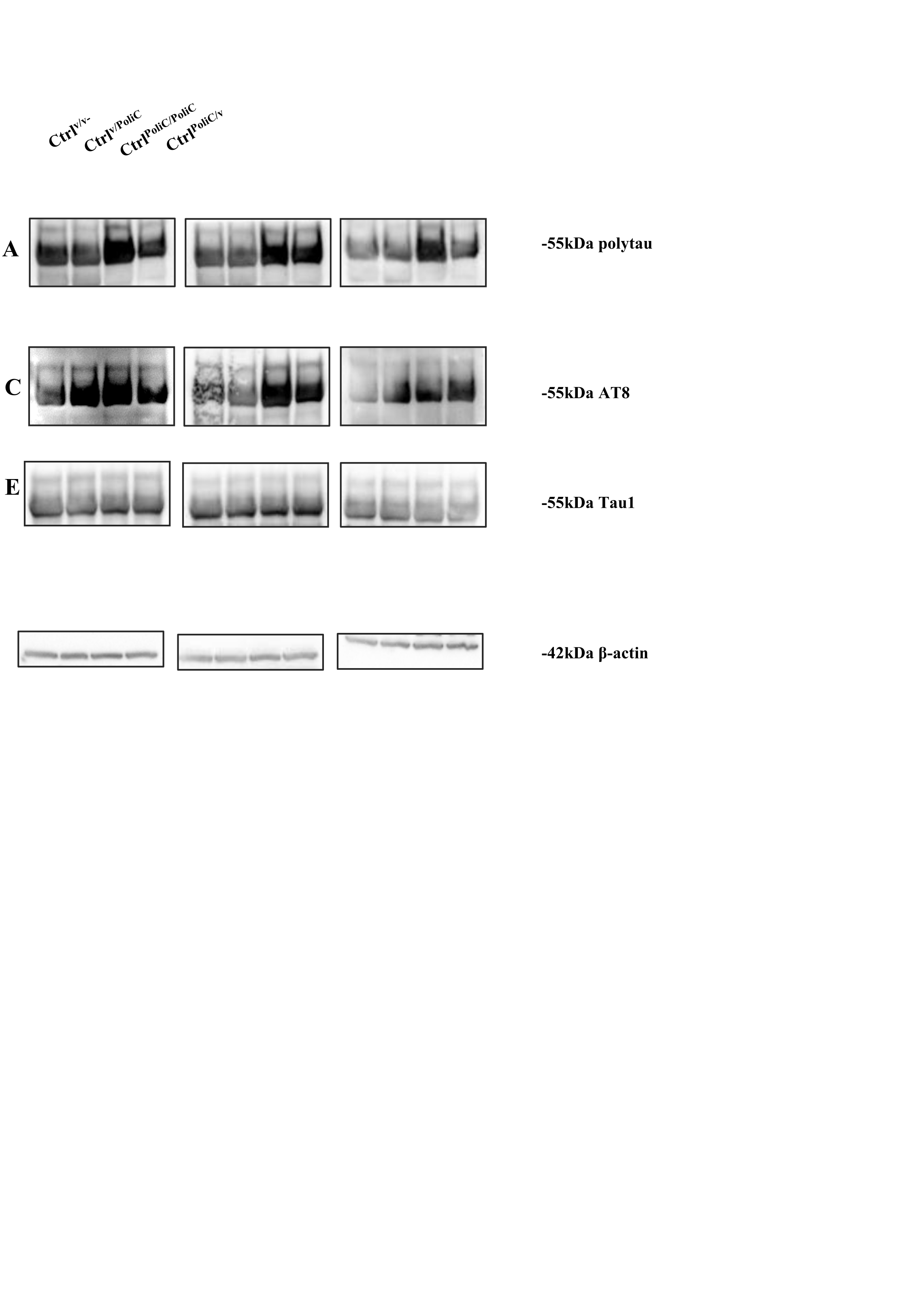

Supplement: Supplementary Figure 6 — Uncropped images of Western blotting analyses of Figure 6. [file Image6.tif]

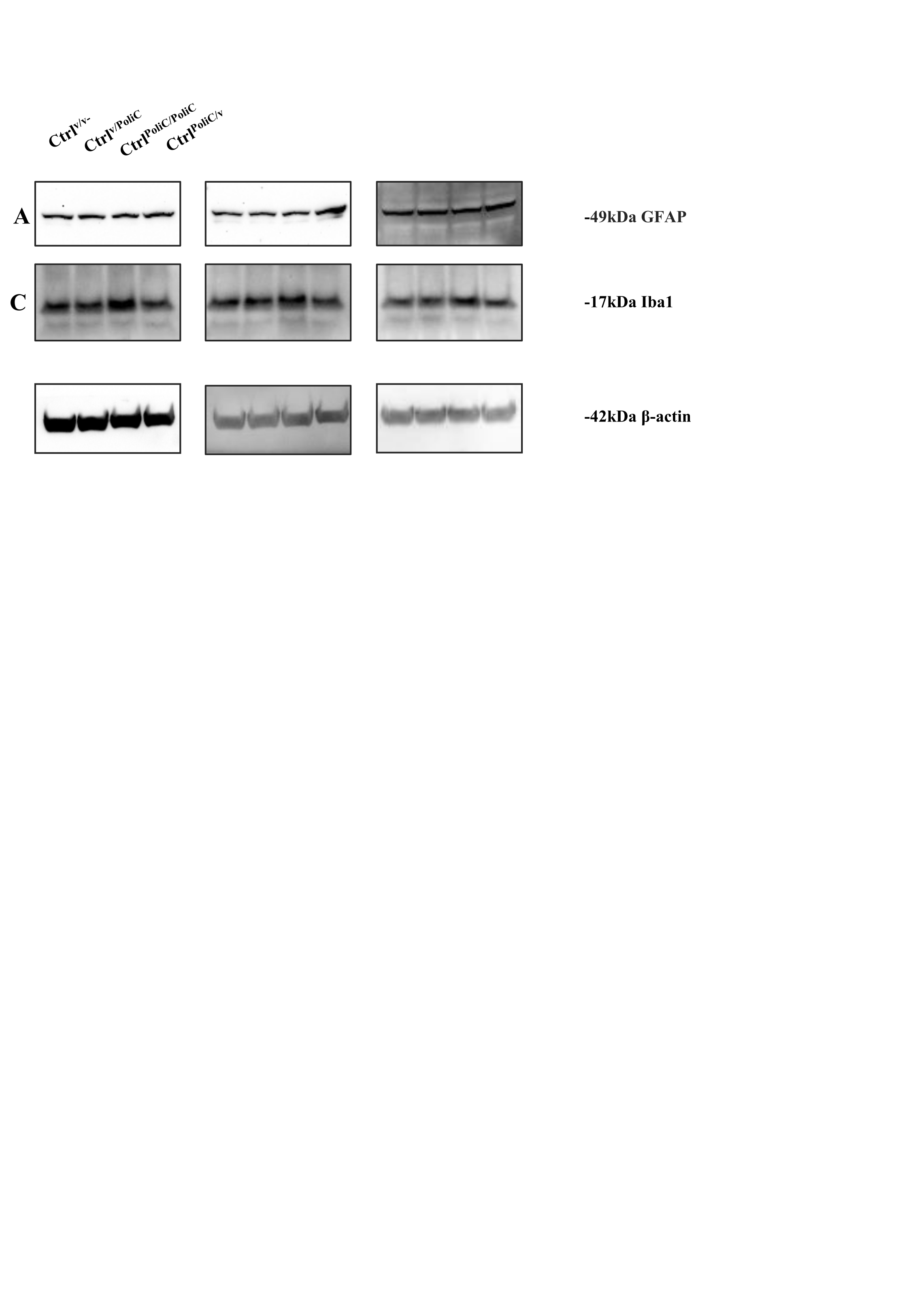

Supplement: Supplementary Figure 7 — Uncropped images of Western blotting analyses of Figure 7. [file Image7.tif]

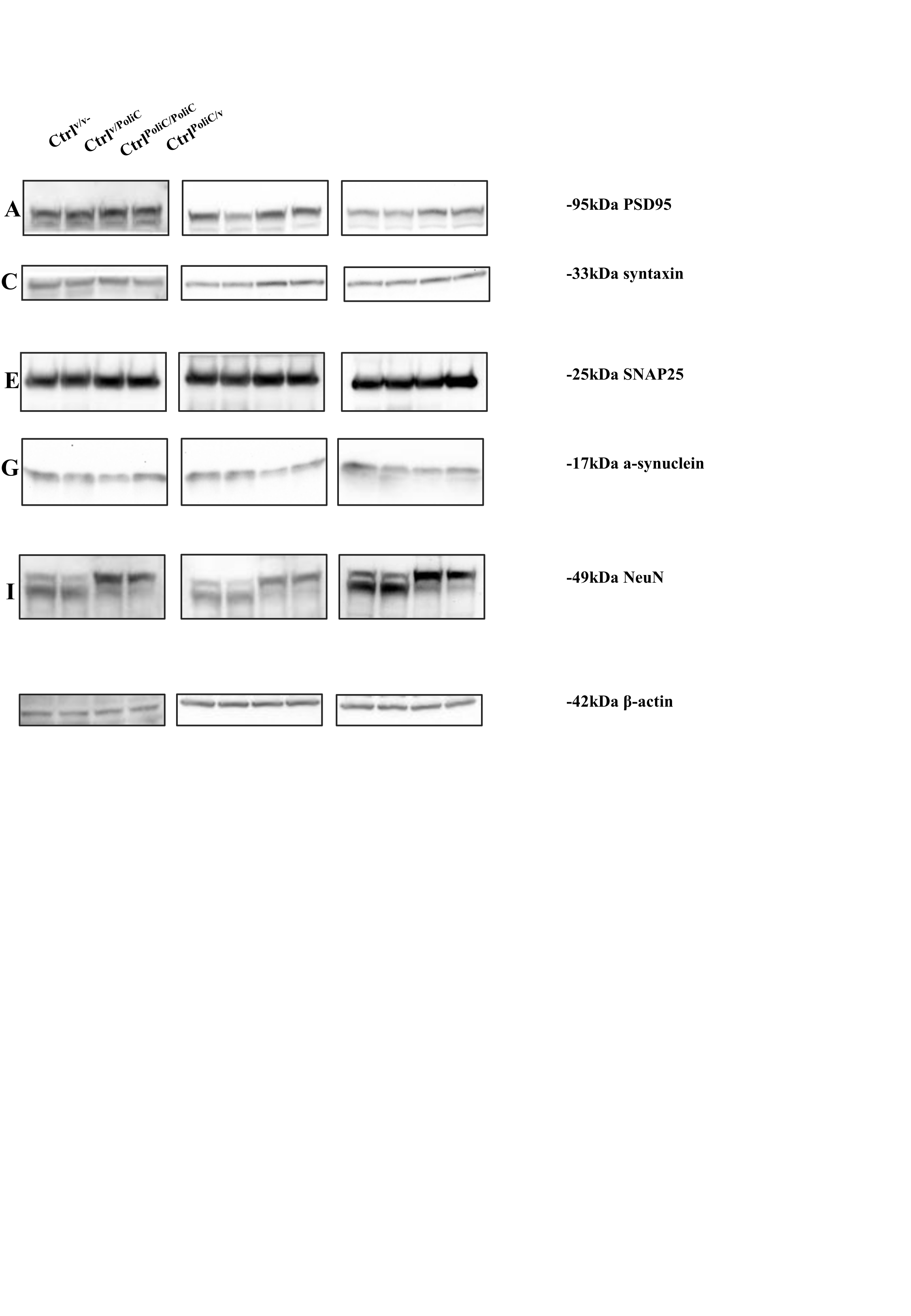

Supplement: Supplementary Figure 8 — Uncropped images of Western blotting analyses of Figure 9. [file Image8.tif]

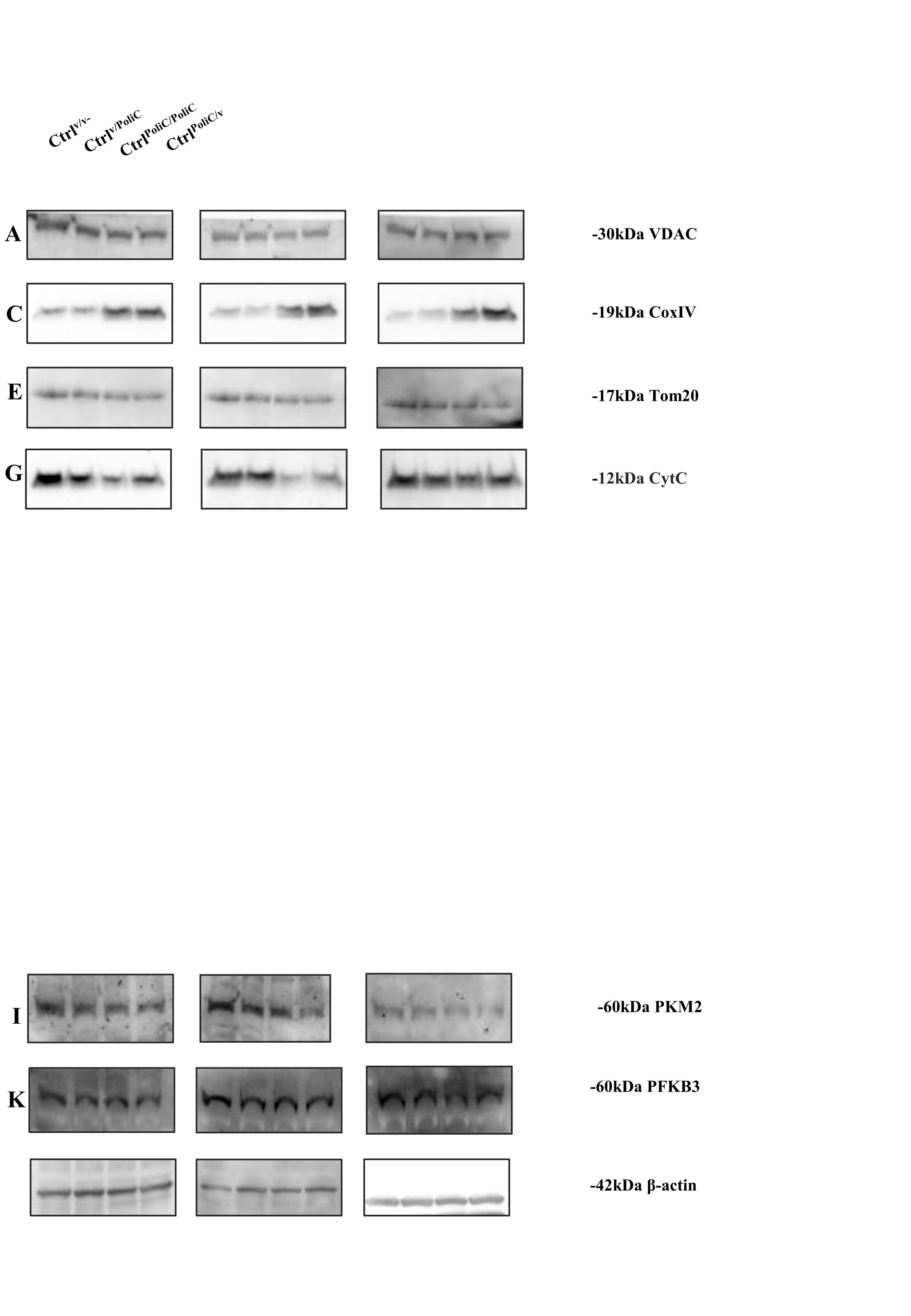

Supplement: Supplementary Figure 9 — Uncropped images of Western blotting analyses of Figure 10. [file Image9.tif]
